# Digital phenotypes of real‐time suicidal ideation: Correlates and consequences

**DOI:** 10.1111/acps.13750

**Published:** 2024-08-26

**Authors:** L. M. M. Kivelä, A. J. W. van der Does, R. Gilissen, Niki Antypa

**Affiliations:** ^1^ Department of Clinical Psychology, Institute of Psychology Leiden University Leiden The Netherlands; ^2^ Leiden University Treatment and Expertise Center (LUBEC) Leiden The Netherlands; ^3^ 113 Suicide Prevention Amsterdam The Netherlands

**Keywords:** passive suicidal ideation, active suicidal ideation, ecological momentary assessment, latent profile analysis, suicide attempts

## Abstract

**Introduction:**

Suicidal ideation variability refers to within‐day fluctuations in suicidal ideation, and has recently been proposed as an indicator of suicide risk. However, not much is known yet about its correlates and clinical relevance.

**Methods:**

We examined characteristics of real‐time suicidal ideation using Ecological Momentary Assessment in 82 individuals with current active suicidal ideation. Data were collected four times daily over 21 days. Latent profile analysis was used to identify subtypes of suicidal ideation. We further examined sociodemographic and clinical correlates of the profiles, and their association with the occurrence of suicide attempts during a 1‐year follow‐up.

**Results:**

We identified three “digital” phenotypes of suicidal ideation that differed on the frequency, intensity and variability of ideation. The profiles were: high frequency, high intensity, moderate variability (Phenotype 1), moderate/high frequency, moderate intensity, high variability (Phenotype 2), and moderate frequency, low intensity, low variability (Phenotype 3). Phenotypes 1 and 2 were associated with a worse clinical profile at baseline (higher suicidal ideation and depressive symptom severity), and increased odds of suicide attempt during follow‐up, compared to Phenotype 3. Phenotype 1 was further characterized by repeated suicidal behavior.

**Conclusions:**

Two phenotypes of real‐time suicidal ideation were identified that appear to confer a higher risk of suicidal behavior in the near future (12 months). These phenotypes were characterized by higher variability of suicidal ideation—and also higher intensity and frequency of ideation. Considering the small sample size, the clinical usefulness of the profiles remains to be demonstrated.

**Significant outcomes**

- Based on real‐time data on suicidal ideation, three distinct phenotypes were identified that were distinguished by their temporal dynamics (incl. intensity, frequency, and variability of suicidal ideation).
- Phenotype 1 was characterized by high frequency/high intensity/moderate variability, Phenotype 2 moderate‐to‐high frequency/moderate intensity/high variability, and Phenotype 3 moderate frequency/low intensity/low variability suicidal ideation.
- Phenotypes 1 and 2 were associated with a worse clinical profile at baseline, and a higher occurrence of suicide attempts over 1‐year of follow‐up.

**Limitations**

- Missing data during the 1‐year follow‐up limited our sample size for the prospective suicide attempt analyses.
- The utility of suicidal ideation phenotypes in predicting suicidal behavior in comparison to other risk factors remains to be established.

## 1 INTRODUCTION

Suicidal ideation can fluctuate greatly in daily life, both between individuals, but also within individuals over time. Recent studies employing real‐time measures (such as Ecological Momentary Assessment, EMA^1^) have illustrated how these moment‐to‐moment changes can be observed in suicidal ideation (see Kivelä et al.^2^ for a review). These studies have illustrated sizeable fluctuations in suicidal ideation over time. For example, among 54 individuals with a recent suicide attempt who completed EMA four times per day over 28 days, approximately one third of suicidal ideation ratings differed from the previous time point by at least one standard deviation, without clear linear changes over time.^3^ Others have presented similar results on the temporal dynamics of suicidal ideation.^4^ These findings illustrate how the transition from low‐ to high‐intensity states may happen within just a few hours.

Identifying those with greater suicidal ideation variability is especially relevant, as indices of variability may provide important information about an individual's risk status. It has been proposed that higher suicidal ideation variability may represent a phenotypic marker for increased suicide risk.^5^, ^6^ Witte et al.^7^, ^8^ previously reported evidence of suicidal ideation variability being related to a prior history of suicide attempts. This finding has since been replicated using real‐time data, whereby those with multiple past suicide attempts (vs. single attempt) exhibited higher suicidal ideation variability.^9^ More recently, temporal variability in suicidal ideation (as measured through EMA during hospitalization) was found to be a better predictor of post‐discharge suicide attempt than baseline sociodemographic or clinical characteristics, or EMA‐measured suicidal ideation intensity.^6^ Explanations for the association between variability and heightened risk status include that individuals may find variability more distressing than stable symptomatology, even when more severe.^8^ Consequently, understanding which individuals are more likely to experience greater variability may be relevant to prevent suicide attempts and mortality.

Individuals with higher (EMA‐measured) mean suicidal ideation scores also have higher variability.^3^, ^5^ However, suicidal ideation variability was found to relate neither to baseline depression nor suicidal ideation severity.^4^ While suicidal ideation variability (as measured with EMA) was found to relate to EMA‐measured depressed mood variability, it did not associate with baseline characteristics, such as general affective lability, or depression or suicidal ideation severity.^9^ Consequently, our understanding of the clinical correlates of suicidal ideation variability is still limited.

The increased application of EMA in suicide research has resulted in a potential new indicator of increased risk: suicidal ideation variability. However, prior research has also identified other predictors of future suicidal behavior, such as the intensity,^10^ frequency,^11^ and peak‐level of ideation.^12^, ^13^ For example, while it is understood that the risk of future suicidal behavior increases as the intensity of ideation increases,^10^ it has also been found that suicidal ideation at its worst point (i.e., peak level) may be a stronger predictor for suicide attempt than its average intensity.^12^, ^13^ Likewise, those with more frequent thoughts about suicide experience heightened risk for future suicidal behavior.^11^ These dynamics are interconnected, and should not be considered in isolation. For example, individuals with very high or low mean intensity of ideation may show less variability due to floor and ceiling effects.^14^

Profiling based on electronically‐collected data on these suicidal ideation dynamics has been called digital phenotyping of suicidal ideation.^2^, ^3^, ^15^ Examining these dynamics, no less than five phenotypes of suicidal ideation were observed in a sample of 51 individuals with a recent suicide attempt: these phenotypes were characterized by low intensity, low variability (Type 1), low intensity, moderate variability (Type 2), moderate intensity, high variability (Type 3), high intensity, low variability (Type 4), and high intensity, high variability (Type 5).^16^ While others have also observed heterogeneity in the short‐term dynamics of suicidal ideation,^4^, ^17^ the suicidal ideation phenotypes have not yet been replicated.

In the present study, we examined suicidal ideation through EMA, four times per day, over 21‐days. Our aim was to examine whether distinct subtypes (i.e., digital phenotypes) would emerge when considering dynamics of real‐time suicidal ideation. Our methodology was based on the prior study by Kleiman et al.,^16^ who created digital phenotypes based on EMA‐measured suicidal ideation intensity (i.e., mean), frequency (i.e., % of non‐zero ratings), peak (i.e., highest score recorded), and variability (as depicted by the within‐person standard deviation, as well as the root mean square of successive differences [RMMSD]). Our aim was to replicate and further extend on this phenotyping approach by considering aspects of both passive and active suicidal ideation (as the previous study was focused on active ideation and intent only), in line with recommendations that comprehensive suicide risk assessments should include both constructs.^18^ Further, we examined which sociodemographic and clinical characteristics were related to these phenotypes, and whether there were differences between the phenotypes in their associated odds of making a suicide attempt during a 1‐year follow‐up.

## 2 METHODS

### 2.1 Participants

Participants (*N* = 82) were adults with a recent (past year) history of a suicide attempt and/or active suicidal ideation (Columbia Suicide Severity Rating Scale [CSSRS]^19^ ≥3, or ≥2 if symptoms were present in the past 2 months). Participants were recruited through referral from collaborating mental health treatment centers, as well as community advertisements. Participants were excluded in case of current bipolar disorder, a psychotic disorder or severe substance dependence; as the present study was designed to examine short‐term (hourly, daily) fluctuations in suicidal ideation, we excluded patients with disorders that are episodic in nature (such as bipolar and psychotic disorders), where such fluctuations may be markedly different depending on episode status. Likewise, extended time periods characterized by substance intoxication may introduce similar confounding effects (for more details see Kivelä et al.^20^). Participants received 20€ compensation after completing the 21‐day EMA period, and a further 30€ after completing the 1‐year follow‐up period, as well as compensation for travel costs (if applicable).

### 2.2 Measures

#### 2.2.1 Baseline characteristics

A custom semi‐structured interview was used to assess participants' age and gender, lifetime history of psychiatric disorders, and current use of psychoactive prescription medication. An adapted version of the CSSRS,^19^ comprised of the first five questions and with additional items included on participants' lifetime history of suicide attempt(s), was used to assess history of suicidal thoughts and behaviors. The M.I.N.I. PLUS International Neuropsychiatric Interview (v. 5)^21^ and the Structured Clinical Interview for DSM‐5 Personality Disorders—Borderline Personality Disorder subscale (SCID‐PD‐BPD)^22^ were used to establish current diagnoses. Self‐report questionnaires assessed symptom severity of psychopathology: the Beck Depression Inventory (BDI‐I),^23^ the Beck Scale for Suicide Ideation (BSSI),^24^ and the Hospital Anxiety and Depression Scale—Anxiety Subscale (HADS‐A).^25^ Participants further completed the Quality of Life Enjoyment and Satisfaction Questionnaire—Short Form (Q‐LES‐Q‐SF),^26^ the Leiden Index of Depression Sensitivity—Revised (LEIDS‐R),^27^ and the State–Trait Anger Expression Inventory—Trait Anger Scale (STAXI‐T).^25^

#### 2.2.2 Ecological momentary assessment (EMA)

Data on momentary suicidal ideation were gathered through 4×/day EMA over 21‐days. Two items were used to measure passive suicidal ideation (“*At the moment… How strong is your desire to live? How strong is your desire to die, or go to sleep and not wake up?*”), and two to measure active ideation (“*At the moment… Do you actually have thoughts of killing yourself? How strong is your intention to act on these thoughts?*”). All items were rated from 0 (None/Not at all) to 10 (Very strong/Very much) (positively worded items were reverse coded). Mean scores were created for each outcome (passive/active suicidal ideation).

#### 2.2.3 Suicide attempts

Data on suicide attempts were gathered through a weekly questionnaire during 12 months. Participants indicated whether they had made a suicide attempt during the previous week (“*Did you make a suicide attempt? Yes/No*”). An aggregate variable was created to indicate whether a participant had a suicide attempt during the 12‐month follow‐up (0 = no, 1 = yes).

### 2.3 Procedure

#### 2.3.1 Intake interview

Participants attended an intake interview during which they received information about the study, and provided written informed consent and data on their sociodemographic and clinical characteristics. After establishing eligibility, personalized safety plans were created for each participant.

#### 2.3.2 Baseline assessment

Following the intake interview (which could be done online or in‐person, depending on the participant's preference), participants received a link to an online questionnaire they were instructed to fill in within 72 h (see *Measures: Baseline characteristics*).

#### 2.3.3 21‐Day EMA

The EMA period commenced the day after the intake interview. Participants received alerts 4×/day through a mobile phone app (*Avicenna [Ethica]*, avicennaresearch.com) on a pseudorandom schedule between 7 a.m. and 10 p.m. Participants had 180 min to fill in the first (i.e., morning) assessment, and 120 min to fill in the remaining assessments during the day; a reminder alert was sent out after 30 min in case the participant had not yet filled in the EMA. Participants could also initiate additional entries at any time (e.g., after missing an entry, or when experiencing high/low suicidal ideation). Eighty‐one participants (99%) completed the 21‐day EMA period (*nb*. prior to withdrawing, the participant who dropped out of the study during the EMA period provided EMA comparable in number to the range observed among the completers (*k* = 16, range among completers *k* = 16–88), and was hence retained in the present analyses).

#### 2.3.4 Weekly questionnaire

After the 21‐day EMA, participants who agreed to continue into the second phase of the study (*n* = 72, 88%) commenced a 12‐month monitoring period during which they filled in a digital questionnaire 1×/week. Each questionnaire was released on a Sunday (using the *Avicenna [Ethica]* app), and participants had 48 h to fill it in; reminder alerts were sent out after 12, 24, and 36 h.

### 2.4 Statistical analysis

We calculated intraclass correlation coefficients (ICC) to quantify within‐ versus between‐person variability, and RMMSD to examine moment‐to‐moment variability in suicidal ideation. The ICC estimates correlation within repeated measures.^28^ Higher ICC scores indicate that a greater amount of the total variation is attributable to between‐personal variation (with 1‐ICC indicating the proportion of within‐person variability). The RMMSD estimates variability over time based on the difference between successive observations within an individual^29^ and has previously been applied to quantify short‐term variability in affect^30^ and suicidal ideation,^17^ as in the previous study by Kleiman et al.^16^ For calculating the RMMSD, we did not remove rows with missing data, ensuring that successive differences were only calculated between two adjacent time points (as also previously done by e.g., Bos et al.^30^).

In IBM SPSS Statistics (v.29), we fitted intercept‐only linear‐mixed models with suicidal ideation as outcome to estimate ICCs. The *psych* package^31^ for R^32^ was used to calculate RMMSD values, and *ggplot2* ^33^ to create time‐series plots to visualize variability. The *mclust* package^34^ was used to perform latent profile analysis (LPA) in order to identify phenotypes of suicidal ideation. We used 10 within‐person characteristics of real‐time suicidal ideation to distinguish the phenotypes: mean of passive (1) and active ideation (2); standard deviation of passive (3) and active ideation (4); peak (i.e., highest score recorded) of passive (5) and active ideation (6); frequency (i.e., percentage of non‐zero ratings) of passive (7) and active suicidal (8); and RMSSD of passive (9) and active ideation (10). These characteristics were based on Kleiman et al.,^16^ but further extended to include estimates of both passive and active suicidal ideation, in line with findings indicating different temporal patterns for different components of ideation.^35^ The within‐person standard deviation and the RMSSD were both used as measures of variability (and collectively referred to as such within the present paper). To further specify, the within‐person standard deviation depicts average within‐person variability over time (i.e., dispersion), while the RMMSD captures the temporal dynamics of short‐term change (i.e., instability).^30^, ^36^ The optimal number of latent profiles was determined based on model fit (the Bayesian Information Criterion [BIC] and the Bootstrapped Likelihood Ratio Test [BLRT] with 1000 resamples) and entropy (i.e., a measure of separation between profiles which estimates the accuracy of classification).^37^ Analyses of variance (ANOVAs) and Chi‐square tests were used to examine differences between phenotypes in suicidal ideation and baseline characteristics. Fisher's exact test was used to examine differences in the occurrence of suicide attempts during follow‐up. Significance was determined at *p* < 0.05.

## 3 RESULTS

### 3.1 Descriptive

The sample (*N* = 82) was predominantly female (77%), with a mean age of 27 (SD = 8.6). Participants on average filled in *M* = 63 (78%) of the scheduled EMA* entries and *M* = 3 additional entries, resulting in *M* = 66 entries completed on average per person. During the 1‐year follow‐up, participants (*n* = 72) on average filled in *M* = 34 (66%) of the weekly questionnaires. Thirty‐six participants had sufficient data to be included in the prospective analyses on suicide attempts, that is, they either reported a suicide attempt (*n* = 7), and/or completed the study assessments up until the end of the 1‐year follow‐up (*n* = 29); participants lost to follow‐up (and who did not report a suicide attempt prior) were excluded in order to ascertain that we would not incorrectly classify any non‐responders as non‐suicide attempters. Those excluded did not significantly differ from those included on age, gender, baseline depressive symptoms, past suicide attempt history or phenotype classification (all *p*'s > 0.05), but had lower baseline suicidal ideation (*M*_included_ = 18.0 vs. *M*_excluded_ = 13.0, *p* = 0.014).

Descriptive statistics for suicidal ideation are presented in Table 3 (correlations and reliability statistics can be found in Supplementary material). Passive suicidal ideation had a higher mean and greater within‐person variability (RMSSD) than active ideation. ICCs indicated that 70% of the variation in passive, and 67% of the variation in active suicidal ideation, was attributable to between‐person variability.

### 3.2 Latent profile analysis of suicidal ideation

We estimated model fit for solutions with 1, 2, 3, 4, 5, and 6 profiles, respectively (Table 1). The BLRT and entropy values indicated improved fit with each successive model. However, the BIC indicated best fit for the model with three profiles. As entropy values may be inflated in overfitted models, we decided to rely on the BIC and chose the three profile solution. This solution also provided group sizes that were approximately equal, whereas the additional profiles only accounted for ≤10% of the sample each (Table 2).

**TABLE 1:** Fit statistics from latent profile analysis (LPA).

	BIC	Entropy	*k* − 1 BLRT
1 Profile	−2880.00	0.00	‐
2 Profile	−2526.03	0.95	751.00, *p* < 0.001
3 Profile	−2481.03	1.55	359.10, *p* < 0.001
4 Profile	−2693.27	1.85	602.47, *p* < 0.001
5 Profile	−2707.86	2.13	377.42, *p* < 0.001
6 Profile	−2793.31	2.42	1001.26, *p* < 0.001

*Note*: BLRT = Bootstrapped likelihood ratio test between two successive models (# profiles − 1).

**TABLE 2:** Profile membership from latent profile analysis (LPA).

	1	2	3	4	5	6
1 Profile	82 (100%)					
2 Profile	52 (63%)	30 (37%)				
3 Profile	20 (24%)	27 (33%)	35 (43%)			
4 Profile	26 (32%)	24 (29%)	26 (32%)	6 (7%)		
5 Profile	25 (30%)	24 (29%)	19 (23%)	5 (6%)	9 (11%)	
6 Profile	17 (21%)	24 (29%)	18 (22%)	6 (7%)	8 (10%)	9 (11%)

*Note*: Individual class probabilities for the three profile solution are included in the Supplementary material.

**FIGURE 1:**
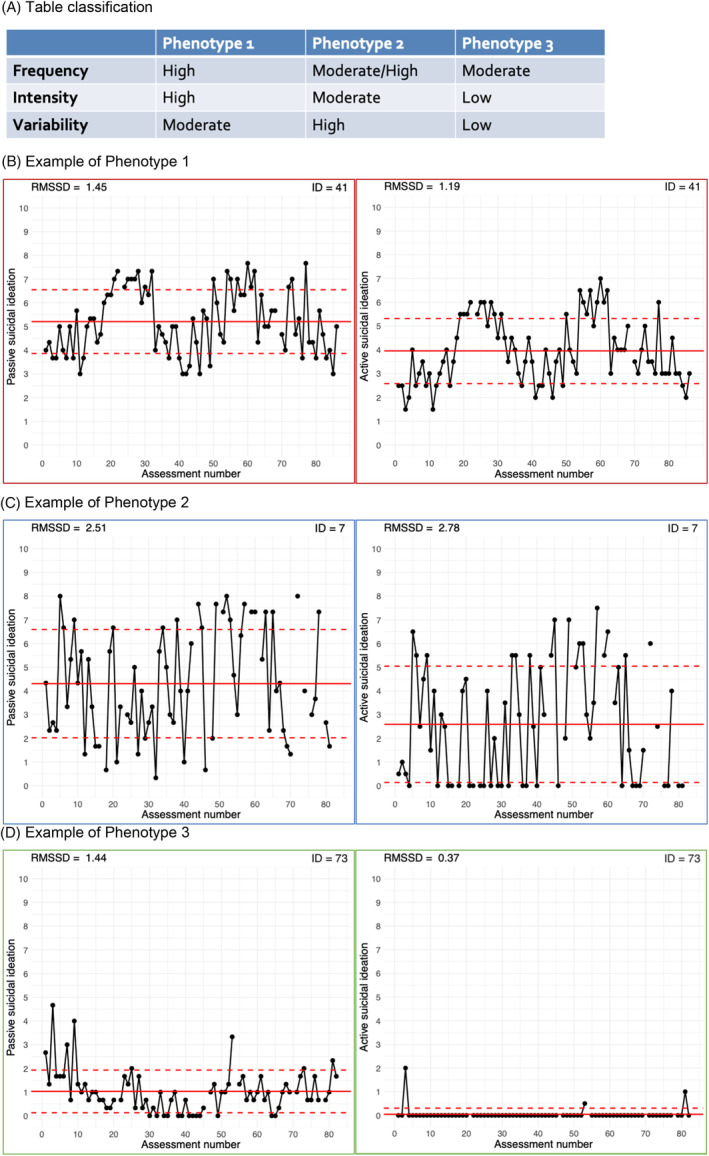
A graphical overview of the defining features of the phenotypes. Time‐series plots indicate the person‐mean (solid red line) and standard deviation around the mean (dashed red lines). The RMSSD (root mean square of successive differences) indicates within‐person variability. Frequency is inferred by scores > zero. Phenotype 1 is represented in red, Phenotype 2 in blue, and Phenotype 3 in green. ID numbers do *not* correspond to participant numbers assigned during data collection.

Differences in suicidal ideation characteristics between the phenotypes are presented in Table 3. Figure 1 presents a graphical overview of the defining features of the phenotypes (A), as well as time‐series plots for example participants from Phenotype 1 (B), Phenotype 2 (C) and Phenotype 3 (D) (see Supplementary material for all time‐series plots).

**TABLE 3:** Characteristics and subtypes of real‐time suicidal ideation.

	Overall (*N* = 82)	Type 1 (*n* = 20)	Type 2 (*n* = 27)	Type 3 (*n* = 35)	ANOVA	*p*‐value
*M*, Passive	2.93	5.25_a_	3.37_b_	1.26_c_	69.29	<0.001
*M*, Active	1.20	3.49_a_	0.89_b_	0.11_c_	66.52	<0.001
SD, Passive	1.21	1.21_a_	1.77_b_	0.77_c_	75.79	<0.001
SD, Active	0.97	1.33_a_	1.46_a_	0.37_b_	43.43	<0.001
Peak, Passive	6.46	8.02_a_	8.01_a_	4.38_b_	51.61	<0.001
Peak, Active	4.51	6.58_a_	6.00_a_	2.17_b_	39.33	<0.001
% Non‐zero, Passive	90.8	99.9_a_	96.2_a_	81.3_b_	7.85	<0.001
% Non‐zero, Active	37.4	95.2_a_	33.6_b_	7.2_c_	308.49	<0.001
RMSSD, Passive	1.36	1.23_a_	1.87_b_	1.04_a_	23.97	<0.001
RMSSD, Active	1.00	1.33_a_	1.49_a_	0.42_b_	26.75	<0.001

*Note*: Subscript letters denote groups that significantly differ from each other based on *p* < 0.05.

Abbreviations: *M*, mean; RMSSD, root mean square of successive differences; SD, standard deviation.

### 3.3 Sociodemographic and clinical correlates of suicidal ideation phenotypes

Differences between the phenotypes on baseline characteristics are presented in Table 4. Phenotype 1 had higher suicidal ideation (BSSI) at baseline compared to Phenotype 2, which in turn had a higher BSSI score than Phenotype 3. Phenotypes 1 and 2 also had higher depressive symptoms, and more cases with current MDD and comorbidity, than Phenotype 3. Further, Phenotype 1 had higher anxiety symptoms and lower quality of life, and more cases with current PTSD, than Phenotype 3. Phenotype 1 had the highest percentage of both people with a past suicide attempt and those with multiple past attempts; however, none of the comparisons on prior suicide attempt history reached statistical significance.

**TABLE 4:** Sociodemographic and clinical correlates of suicidal ideation subtypes.

	Overall (*N* = 82)	Type 1 (*n* = 20)	Type 2 (*n* = 27)	Type 3 (*n* = 35)	ANOVA/Chi‐square	*p*‐value
Age	27.2	27.5_a_	25.5_a_	28.3_a_	0.88	0.420
Gender, Female	63 (77%)	16 (80%)_a_	18 (67%)_a_	29 (83%)_a_	2.39	0.302
Diagnosis						
MDD	41 (50%)	14 (70%)_a_	19 (70%)_a_	8 (23%)_b_	17.20	<0.001
Anxiety disorders	47 (57%)	13 (65%)_a_	18 (67%)_a_	16 (46%)_a_	2.91	0.234
PTSD	18 (22%)	8 (40%)_a_	7 (26%)_ab_	3 (9%)_b_	7.40	0.025
BPD	12 (15%)	2 (10%)_a_	6 (22%)_a_	4 (11%)_a_	1.79	0.408
OCD	7 (9%)	0 (0%)_a_	3 (11%)_a_	4 (11%)_a_	2.52	0.284
ADHD	10 (12%)	2 (10%)_a_	5 (19%)_a_	3 (9%)_a_	1.44	0.486
ASD	14 (17%)	6 (30%)_a_	4 (15%)_a_	4 (11%)_a_	3.26	0.197
Comorbidity	57 (70%)	17 (85%)_a_	23 (85%)_a_	17 (49%)_b_	11.66	0.003
Symptom severity						
BSSI	15.3	22.5_a_	15.8_b_	10.5_c_	15.32	<0.001
BDI	25.5	32.3_a_	27.3_a_	19.9_b_	13.38	<0.001
HADS‐A	11.5	13.3_a_	11.5_ab_	10.6_b_	3.49	0.036
Q‐LES‐Q‐SR	43.0	37.6_a_	42.3_ab_	47.0_b_	6.75	0.002
LEIDS‐R	65.5	66.9_a_	65.8_a_	63.2_a_	0.32	0.730
STAXI‐T	19.4	18.6_a_	19.5_a_	19.7_a_	0.23	0.798
Medication						
Antidepressants	33 (40%)	9 (45%)_a_	10 (37%)_a_	14 (40%)_a_	0.304	0.859
Anxiolytics/Sedatives	20 (24%)	6 (30%)_a_	5 (19%)_a_	9 (25%)_a_	0.88	0.644
Stimulants	10 (12%)	1 (5%)	5 (19%)_a_	4 (11%)_a_	1.99	0.369
Suicide attempt history						
Yes	35 (42%)	10 (50%)_a_	11 (41%)_a_	14 (40%)_a_	0.58	0.747
Yes, multiple	24 (29%)	8 (40%)_a_	9 (33%)_a_	7 (20%)_a_	4.98	0.083
Recent (past 12 months)	17 (21%)	5 (25%)_a_	6 (22%)_a_	6 (17%)_a_	0.35	0.840

*Note*: Subscript letters denote groups that significantly differ from each other based on *p* < 0.05.

Abbreviations: ADHD, attention deficit hyperactivity disorder; ASD, autism spectrum disorder; BDI, Beck Depression Inventory; BPD, borderline personality disorder; BSSI, Beck Scale for Suicide Ideation; Comorbidity, i.e., more than one current diagnosis; HADS‐A, Hamilton Anxiety and Depression Scale—Anxiety Subscale; LEIDS‐R, Leiden Index of Depression Sensitivity—Revised; MDD, major depressive disorder; OCD, obsessive compulsive disorder; PTSD, post‐traumatic stress disorder; Q‐LES‐Q‐SR, Quality of Life Enjoyment and Satisfaction Questionnaire—Short Form; STAXI‐T, State–Trait Anger Expression Inventory—Trait Anger Scale.

### 3.4 Risk of future suicide attempt

**FIGURE 2:**
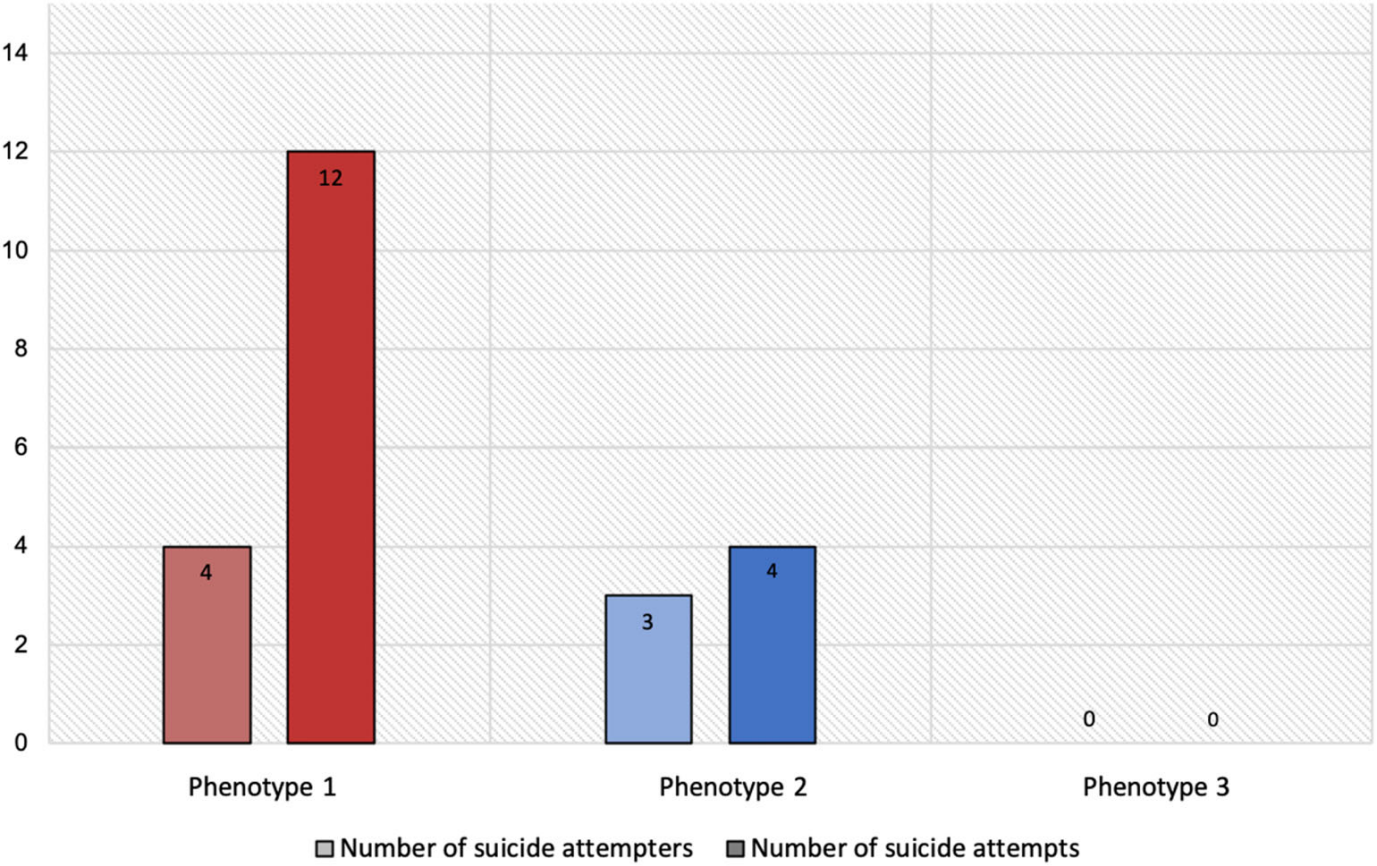
Number of suicide attempters and attempts as a function of phenotype.

Follow‐up data (*n* = 36) was available for 55% of individuals for Phenotype 1, 41% for Phenotype 2, and 40% for Phenotype 3; phenotype categorization was not a significant determinant of exclusion from the follow‐up analyses (*p* = 0.515). During the subsequent 1‐year, seven participants reported a total of 16 suicide attempts (*Med* = 2, *Range* 1–5 attempts/person). Participants with Phenotypes 1 and 2 were significantly more likely to make a suicide attempt during follow‐up than those with Phenotype 3 (with no difference between Phenotypes 1 and 2), based on Fisher's exact test (*p* = 0.040, Cramer's *V* = 0.40). Further, Phenotype 1 was specifically characterized by repeat suicidal behavior, with four participants in Phenotype 1 (*n* = 11) accounting for 12 suicide attempts, and three participants in Phenotype 2 (*n* = 11) accounting for four attempts (with no suicide attempts in Phenotype 3, *n* = 14) (Figure 2). In comparison, those with a past suicide attempt history (which is generally considered to be the best predictor of future suicidal behavior) were also significantly more likely to make a suicide attempt during follow‐up (*p* = 0.002, Cramer's *V* = 0.52).

An exploratory analysis of the 17 participants with a past suicide attempt history revealed that the distribution across phenotypes was 7 (Phenotype 1), 7 (Phenotype 2), and 3 (Phenotype 3). The number of participants with a suicide attempt during follow‐up was 4 (Phenotype 1), 3 (Phenotype 2), and 0 (Phenotype 3). Hence, 50% of those with a past suicide attempt history within Phenotypes 1 and 2 had a repeat attempt, compared to 0% of those within Phenotype 3.

## 4 DISCUSSION

In the present study, we used EMA data to identify digital phenotypes of suicidal ideation. A three‐profile solution provided the best fit. We also found that these phenotypes were associated with distinct clinical profiles at baseline and different odds of making a suicide attempt during a 1‐year follow‐up, although the latter finding warrants replication in larger samples.

The first attempt to apply digital phenotyping to electronically‐collected data on suicidal ideation was based on a sample of 51 individuals with a recent suicide attempt.^16^ Five phenotypes were identified, predominantly distinguished by differences in the intensity and variability of ideation. Our analyses indicated the presence of three phenotypes that partly overlap with the previously identified profiles. Our Phenotype 2 roughly corresponds to the previously identified Type 3 (moderate mean, high variability), and our Phenotype 3 to the previously identified Type 1 (low mean, low variability). The remaining two phenotypes with low numbers of participants (*n* < 10) in the Kleiman et al.^16^ study instead appear to merge with the three identified phenotypes in our sample (see Supplementary material for a graphical overview). It should also be noted that in contrast to Kleiman et al.,^16^ we considered aspects of passive and active suicidal ideation separately, whereas they predominantly focused on active ideation (incl. active ideation, intent, and acquired capability). Differences between the categorizations may therefore be explained by the inclusion of items specifically estimating passive ideation. However, it is also possible that simply with the higher number of predictors included, our model converged better with fewer clusters. Indeed, the entropy values of the LPA solutions were fairly large, which can indicate overfitting. However, individual class probabilities of the final three profile solution were high (0.88–1.00), indicating that the estimated probability that a given individual belongs to the group they were assigned to was between 88% and 100% (see Supplementary material).

The idea of establishing suicidal ideation phenotypes has existed long before the advent of real‐time monitoring studies. For example, two subtypes of suicidal ideation have been proposed, characterized by variable versus stable ideation.^38^ Integrating more comprehensive data on the temporal dynamics of suicidal ideation, our findings as well as those of Kleiman et al.,^16^ illustrate that even more distinct subtypes of suicidal ideation may emerge. Further, these subtypes are differentiated not only by variability, but also other dynamic characteristics of suicidal ideation, such as frequency and intensity.

Examination of baseline characteristics indicated worse clinical profiles for Phenotypes 1 and 2, most prominently higher suicidal ideation and depressive symptom severity, and more comorbidity, compared to Phenotype 3. Furthermore, Phenotype 1 had the highest number of both suicide attempters and those with multiple past attempts, increased anxiety levels and more patients with a PTSD diagnosis; however, these comparisons were not significantly different from estimates in Phenotype 2. Hence, it appears that both Phenotype 1 and 2 may capture those patients with more chronic, and comorbid symptomatology (as indicated by higher symptom severity on longitudinal symptom measures, as well as a higher incidence of psychiatric disorders and comorbidity); this observation needs further verification in future research.

When examining the prospective occurrence of suicide attempts over 1 year, we found Phenotypes 1 and 2 to be at a significantly higher risk of future suicidal behavior compared to Phenotype 3 (effect size *V* = 40). In comparison, past suicide attempt history had an effect size of *V* = 52, indicating that both are strong predictors^39^ of future suicidal behavior. Further, Phenotype 1 was specifically associated with repeat suicidal behavior (i.e., multiple attempts). It should be noted that a history of suicide attempt more strongly predicted future suicidal behavior than the digital phenotypes. Future studies may investigate whether the combination of past history and phenotype indicators further improves prediction. In our sample, all participants who made a suicide attempt during follow‐up had a past suicide attempt history. Suicide attempt history alone may therefore have limited specificity in identifying those individuals with a past suicide attempt history that are at *lower* risk, especially in the near term (identified as Phenotype 3 in our sample). Predicting re‐attempt among those with a past suicide attempt history is difficult, as other established predictors (such as sociodemographic characteristics and psychiatric comorbidity^40^, ^41^) are rather general predictors of not only re‐attempt, but also index attempt, and initial suicidal ideation.^10^ Hence, risk management among past suicide attempters remains a distinctive challenge. Further, identifying those individuals at risk of repeat suicidal behavior is crucial, as the number of past suicide attempts significantly increases the risk of completed suicide.^42^ Our findings indicate that real‐time suicidal ideation characteristics may aid in identifying not only those at risk of future suicidal behavior (Phenotypes 1 and 2), but specifically those at risk of repeat attempts (Phenotypes 1). This is especially relevant, as Phenotypes 1 and 2 (which were both characterized by a worse clinical profile at baseline) may not readily be differentiated by patient characteristics alone.

Our findings suggest that indices of real‐time suicidal ideation may provide important information about an individual's risk status. Specifically, suicidal ideation variability may represent a marker for increased suicide risk.^7^, ^8^ Our Phenotypes 1 and 2 were associated with higher variability and increased risk of suicide attempt. However, we observed no further differences between Phenotypes 1 and 2, although we expected that Phenotype 2 (with the highest variability) would confer the highest risk. Further, Phenotypes 1 and 2 were also associated with higher intensity and frequency of ideation, indicating that variability should not be considered in isolation. Hence it seems that both high intensity ideation together with moderate variability, as well as moderate intensity ideation with high variability, may confer increased risk. Our results also partly align with the finding that suicidal ideation variability was a risk factor for making a suicide attempt in the month following discharge from inpatient care.^6^ Here, we demonstrate that digital phenotypes (including variability) may predict risk during the next 12 months. An exploratory analysis suggests that the prediction may be improved by considering both past behavior and current phenotype.

Future research should further examine outcomes related to suicidal ideation phenotypes. For example, it has been suggested that those with more variable suicidal ideation are more impacted by stressful life events, and may represent more “impulsive” suicide attempters.^43^ Therefore, future research should consider how these phenotypes interact with other risk factors (such as patient characteristics and environmental stressors) in their associations with suicidal behavior. It has been proposed that phenotyping of suicidal ideators may pave the way for more personalized treatment,^44^ but such interventions require further knowledge on these interactions. Methodological considerations for future research include establishing more standardized, and reliable, protocols to quantify variability in suicidal ideation. While the RMSSD (or the mean square of successive differences, MSSD) is the most frequently used measure to indicate variability in EMA‐measured suicidal ideation (see e.g., Hallensleben et al.,^4^ Kleiman et al.,^3^, ^16^ Oquendo et al.,^5^ Peters et al.,^9^ Rizk et al.,^17^ Wang et al.,^6^ and Witte et al.^7^, ^8^), and is also frequently used in similar EMA designs to quantify variability in affect (see e.g., Bos et al.^30^), there are some limitations to how it is currently used in the EMA‐suicide literature. For example, the RMSSD assumes equally spaced observations—an assumption that is violated both by the present study (due to the inclusion of night‐to‐morning time jumps) as well as each of the prior studies mentioned, none of which (reported that they) accounted for transitions between days. We therefore opted to follow the same methodology in order to establish comparability with our results and that of prior studies focusing on suicidal ideation assessments using EMA. However, future research should account for different time lags in their RMSSD calculations, as previously done in other EMA research (see e.g., Ebner‐Priemer et al.,^45^ Jahng et al.,^46^ and Sperry and Kwapil^47^).

A number of limitations should be considered. Our approach was exploratory, and we did not correct for multiple testing. The number and characteristics of the digital phenotypes may be dependent on population and sample size. Replication of these findings in larger and more representative samples is needed, in order to account for the diversity of individuals experiencing suicidal ideation. This way, the phenotypes that exhibit the most consistency across samples may be identified, prior to drawing further conclusions about their clinical relevance. Further, within our 1‐year monitoring, we included an item only on suicide attempts, and did not inquire about related, preparatory behaviors (such as planning, or obtaining means). However, such behaviors may represent important indicators of risk. Future studies employing similar longer‐term repeated assessments may consider incorporating such dimensions in their assessments. This would also allow to test the hypothesis that those with more variable suicidal ideation transition more impulsively to attempt (as proposed by Bostwick et al.^43^).

In conclusion, digital phenotypes of real‐time suicidal ideation appear to be associated with different clinical profiles and risk of future suicidal behavior. Profiles associated with an increased occurrence of suicide attempts were characterized by higher variability in suicidal ideation—but also by higher intensity and frequency. Comprehensive suicide risk assessments may benefit from considering multiple characteristics of ideation; our findings show that intensity levels remain a crucial factor to assess, and that variability and frequency can further add important information to clinical assessments. It remains to be examined whether phenotypes significantly add predictive value when considered in tandem with other established risk factors, in order to further elucidate on the utility of such phenotyping.

## AUTHOR CONTRIBUTIONS

N.W.O. had no role in the study design, collection, analysis or interpretation of the data, writing the manuscript, or the decision to submit the article for publication.

## FUNDING INFORMATION

Funding for this study was provided by the Netherlands Organisation for Scientific Research (N.W.O.) Research Talent Grant 406.18.521. N.W.O. had no role in the study design, collection, analysis or interpetation of the data, writing of the manuscript, or the decision to submit the article for publication.

## CONFLICT OF INTEREST STATEMENT

The authors declare no conflicts of interest.

### PEER REVIEW

The peer review history for this article is available at https://www.webofscience.com/api/gateway/wos/peer‐review/10.1111/acps.13750.

## ETHICS STATEMENT

The study was approved by the Medical Ethics Committee—Leiden, Den Haag, Delft (METC‐LDD) on 24.4.2020 with dossier number NL71510.058.19.

## CONSENT STATEMENT

All participants provided written informed consent.

## Supporting information


Supplementary material S1:

## Data Availability

The dataset reported and analyzed in this paper is available in the Data Archiving and Networked Services (DANS) repository following study completion. Data requests may also be submitted to the corresponding author.

## Footnotes

* Participants filled in four daily assessments per day for the first 20 days, as well as a final morning assessment on day 21, resulting in a total of 81 scheduled prompts.

## References

[1] Shiffman S , Stone AA , Hufford MR . Ecological momentary assessment. Annu Rev Clin Psychol. 2008;4(1):1‐32. doi:10.1146/annurev.clinpsy.3.022806.091415 18509902

[2] Kivelä L , van der Does WAJ , Riese H , Antypa N . Don't miss the moment: a systematic review of ecological momentary assessment in suicide research. Front Digit Health. 2022;4:876595. doi:10.3389/fdgth.2022.876595 35601888 PMC9120419

[3] Kleiman EM , Turner BJ , Fedor S , Baele EE , Huffman JC , Nock MK . Examination of real‐time fluctuations in suicidal ideation and its risk factors: results from two ecological momentary assessment studies. J Abnorm Psychol. 2017;126(6):726‐738. doi:10.1037/abn0000273.supp 28481571

[4] Hallensleben N , Spangenberg L , Forkmann T , et al. Investigating the dynamics of suicidal ideation: preliminary findings from a study using ecological momentary assessments in psychiatric inpatients. Crisis. 2018;39(1):65‐69. doi:10.1027/0227-5910/a000464 28468557

[5] Oquendo MA , Galfalvy HC , Choo TH , et al. Highly variable suicidal ideation: a phenotypic marker for stress induced suicide risk. Mol Psychiatry. 2020;26:5079‐5086. doi:10.1038/s41380-020-0819-0 32576966 PMC7755748

[6] Wang SB , Coppersmith DDL , Kleiman EM , et al. A pilot study using frequent inpatient assessments of suicidal thinking to predict short‐term postdischarge suicidal behavior. JAMA Netw Open. 2021;4(3):e210591. doi:10.1001/jamanetworkopen.2021.0591 33687442 PMC7944382

[7] Witte TK , Fitzpatrick KK , Joiner TE , Schmidt NB . Variability in suicidal ideation: a better predictor of suicide attempts than intensity or duration of ideation? J Affect Disord. 2005;88(2):131‐136. doi:10.1016/j.jad.2005.05.019 16054227

[8] Witte TK , Fitzpatrick KK , Warren KL , Schatschneider C , Schmidt NB . Naturalistic evaluation of suicidal ideation: variability and relation to attempt status. Behav Res Ther. 2006;44(7):1029‐1040. doi:10.1016/j.brat.2005.08.004 16188225

[9] Peters EM , Dong LY , Thomas T , et al. Instability of suicidal ideation in patients hospitalized for depression: an exploratory study using smartphone ecological momentary assessment. Arch Suicide Res. 2020;26(1):56‐69. doi:10.1080/13811118.2020.1783410 32654657

[10] Nock MK , Borges G , Bromet EJ , et al. Cross‐national prevalence and risk factors for suicidal ideation, plans and attempts. Br J Psychiatry. 2008;192(2):98‐105. doi:10.1192/bjp.bp.107.040113 18245022 PMC2259024

[11] Chang EC , Chang OD . Development of the frequency of suicidal ideation inventory: evidence for the validity and reliability of a brief measure of suicidal ideation frequency in a college student population. Cogn Ther Res. 2016;40(4):549‐556. doi:10.1007/s10608-016-9758-0

[12] Beck AT , Brown GK , Steer RA , Dahlsgaard KK , Grisham JR . Suicide ideation at its worst point: a predictor of eventual suicide in psychiatric outpatients. Suicide Life Threat Behav. 1999;29(1):1‐9. 10322616

[13] Law KC , Jin HM , Anestis MD . The intensity of suicidal ideation at the worst point and its association with suicide attempts. Psychiatry Res. 2018;269:524‐528. doi:10.1016/j.psychres.2018.08.094 30195747

[14] Bos FM . Ecological Momentary Assessment as a Clinical Tool in Psychiatry: Promises, Pitfalls, and Possibilities [Doctoral Dissertation]. University of Groningen; 2021. 36734692

[15] Ballard ED , Gilbert JR , Wusinich C , Zarate CA . New methods for assessing rapid changes in suicide risk. Front Psych. 2021;12:598434. doi:10.3389/fpsyt.2021.598434 PMC7870718 33574775

[16] Kleiman EM , Turner BJ , Fedor S , et al. Digital phenotyping of suicidal thoughts. Depress Anxiety. 2018;35(7):601‐608. doi:10.1002/da.22730 29637663

[17] Rizk MM , Choo TH , Galfalvy H , et al. Variability in suicidal ideation is associated with affective instability in suicide attempters with borderline personality disorder. Psychiatry. 2019;82:173‐178. 31013205 10.1080/00332747.2019.1600219 PMC6554039

[18] Wastler HM , Khazem LR , Ammendola E , et al. An empirical investigation of the distinction between passive and active ideation: understanding the latent structure of suicidal thought content. Suicide Life Threat Behav. 2023;53(2):219‐226. doi:10.1111/sltb.12935 36504400

[19] Posner K , Brown GK , Stanley B , et al. The Columbia–Suicide Severity Rating Scale: initial validity and internal consistency findings from three multisite studies with adolescents and adults. Am J Psychiatry. 2011;168(12):1266‐1277. doi:10.1176/appi.ajp.2011.10111704 22193671 PMC3893686

[20] Kivelä LMM , Fiß F , van der Does W , Antypa N . Examination of acceptability, feasibility, and iatrogenic effects of ecological momentary assessment (EMA) of suicidal ideation. Assessment. 2023;31:1292‐1308. doi:10.1177/10731911231216053 38098238 PMC11292966

[21] Sheehan DV , Lecrubier Y , Sheehan KH , et al. The mini‐international neuropsychiatric interview (M.I.N.I.): the development and validation of a structured diagnostic psychiatric interview for DSM‐IV and ICD‐10. J Clin Psychiatry. 1998;59(Suppl 20):22‐57. 9881538

[22] First MB . Structured clinical interview for the *DSM* (SCID). The Encyclopedia of Clinical Psychology. John Wiley & Sons, Inc; 2015:1‐6. doi:10.1002/9781118625392.wbecp351

[23] Beck AT . An inventory for measuring depression. Arch Gen Psychiatry. 1961;4(6):561‐571. doi:10.1001/archpsyc.1961.01710120031004 13688369

[24] Beck AT , Kovacs M , Weissman A . Assessment of suicidal intention: the scale for suicide ideation. J Consult Clin Psychol. 1979;47(2):343‐352. 469082 10.1037//0022-006x.47.2.343

[25] Zigmond AS , Snaith RP . The hospital anxiety and depression scale. Acta Psychiatr Scand. 1983;67(6):361‐370. doi:10.1111/j.1600-0447.1983.tb09716.x 6880820

[26] Endicott J , Nee J , Harrison W , Blumenthal R . Quality of life enjoyment and satisfaction questionnaire: a new measure. Psychopharmacol Bull. 1993;29(2):321‐326. 8290681

[27] Solis E , Antypa N , Conijn JM , Kelderman H , Van der Does W . Psychometric properties of the Leiden index of depression sensitivity (LEIDS). Psychol Assess. 2017;29(2):158‐171. doi:10.1037/pas0000326 27148789

[28] Liljequist D , Elfving B , Skavberg Roaldsen K . Intraclass correlation—a discussion and demonstration of basic features. PLoS One. 2019;14(7):e0219854. doi:10.1371/journal.pone.0219854 31329615 PMC6645485

[29] von Neumann J , Kent RH , Bellinson HR , Hart BI . The mean square successive difference. Ann Math Stat. 1941;12(2):153‐162. doi:10.1214/aoms/1177731746

[30] Bos EH , de Jonge P , Cox RFA . Affective variability in depression: revisiting the inertia–instability paradox. Br J Psychol. 2019;110(4):814‐827. doi:10.1111/bjop.12372 30588616 PMC6899922

[31] Revelle W . Psych: Procedures for Psychological, Psychometric, and Personality Research [R package version 2.3.9]. Northwestern University; 2023. https://cran.r-project.org/web/packages/psych/index.html

[32] R Core Team . R: A Language and Environment For Statistical Computing. R Foundation for Statistical Computing; 2016. https://www.R-project.org/

[33] Wickham H . ggplot2: Elegant Graphics for Data Analysis. Springer‐Verlag; 2016. https://ggplot2.tidyverse.org/

[34] Scrucca L , Fop M , Murphy TB , Raftery AE . Mclust 5: clustering, classification and density estimation using gaussian finite mixture models. R J. 2016;8(1):289. doi:10.32614/RJ-2016-021 27818791 PMC5096736

[35] Oakey‐Frost N , Moscardini EH , Cowan T , Cohen A , Tucker RP . The temporal dynamics of wish to live, wish to die, and their short‐term prospective relationships with suicidal desire. Behav Ther. 2023;54(3):584‐594. doi:10.1016/j.beth.2022.12.011 37088512

[36] Dejonckheere E , Mestdagh M , Houben M , et al. Complex affect dynamics add limited information to the prediction of psychological well‐being. Nat Hum Behav. 2019;3(5):478‐491. doi:10.1038/s41562-019-0555-0 30988484

[37] Sinha P , Calfee CS , Delucchi KL . Practitioner's guide to latent class analysis: methodological considerations and common pitfalls. Crit Care Med. 2021;49(1):e63‐e79. doi:10.1097/CCM.0000000000004710 33165028 PMC7746621

[38] Bernanke JA , Stanley BH , Oquendo MA . Toward fine‐grained phenotyping of suicidal behavior: the role of suicidal subtypes. Mol Psychiatry. 2017;22(8):1080–1081. doi:10.1038/mp.2017.123 28607457 PMC5785781

[39] Kim H‐Y . Statistical notes for clinical researchers: chi‐squared test and Fisher's exact test. Restorat Dent Endodont. 2017;42(2):152‐155. doi:10.5395/rde.2017.42.2.152 PMC5426219 28503482

[40] Irigoyen M , Porras‐Segovia A , Galván L , et al. Predictors of re‐attempt in a cohort of suicide attempters: a survival analysis. J Affect Disord. 2019;247:20‐28. doi:10.1016/j.jad.2018.12.050 30640026

[41] Parra‐Uribe I , Blasco‐Fontecilla H , Garcia‐Parés G , et al. Risk of re‐attempts and suicide death after a suicide attempt: a survival analysis. BMC Psychiatry. 2017;17(1):163. doi:10.1186/s12888-017-1317-z 28472923 PMC5415954

[42] Azcárate‐Jiménez L , López‐Goñi JJ , Goñi‐Sarriés A , Montes‐Reula L , Portilla‐Fernández A , Elorza‐Pardo R . Repeated suicide attempts: a follow‐up study. Actas Espan Psiquiatr. 2019;47(4):127‐136. 31461152

[43] Bostwick JM , Pabbati C , Geske JR , McKean AJ . Suicide attempt as a risk factor for completed suicide: even more lethal than we knew. Am J Psychiatry. 2016;173(11):1094‐1100. doi:10.1176/appi.ajp.2016.15070854 27523496 PMC5510596

[44] Barrigon ML , Courtet P , Oquendo M , Baca‐García E . Precision medicine and suicide: an opportunity for digital health. Curr Psychiatry Rep. 2019;21(12):131. doi:10.1007/s11920-019-1119-8 31776806

[45] Ebner‐Priemer UW , Eid M , Kleindienst N , Stabenow S , Trull TJ . Analytic strategies for understanding affective (in)stability and other dynamic processes in psychopathology. J Abnorm Psychol. 2009;118(1):195‐202. 19222325 10.1037/a0014868

[46] Jahng S , Wood PK , Trull TJ . Analysis of affective instability in ecological momentary assessment: indices using successive difference and group comparison via multilevel modeling. Psychol Methods. 2008;13(4):354‐375. 19071999 10.1037/a0014173

[47] Sperry SH , Kwapil TR . Comparing static and dynamic measures of affect intensity and affect lability: do they measure the same thing? Motivat Emot. 2020;44:870‐879.

