# Supplementary material for: Digital phenotypes of real‐time suicidal ideation: Correlates and consequences
Source: Acta Psychiatr Scand. 2024 Aug 26;151(3):375–87. doi: 10.1111/acps.13750 (PMC11787920; doi:10.1111/acps.13750)
Supplement: Supplementary file 1 — Supplementary material S1: [file ACPS-151-375-s001.docx]

**Supplementary material (Kivelä et al. 2024)**

**Figure S1.** Variability in passive suicidal ideation

**
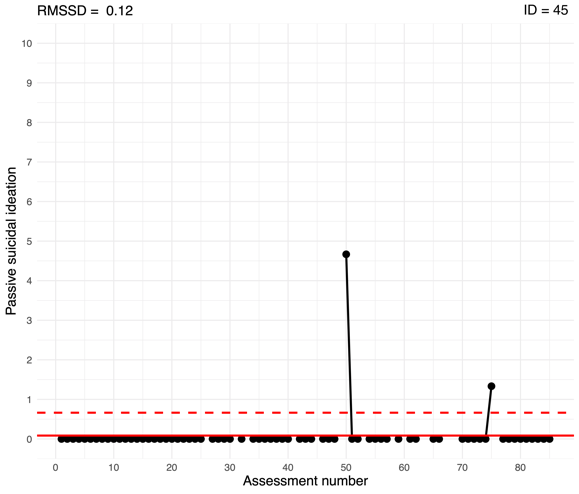

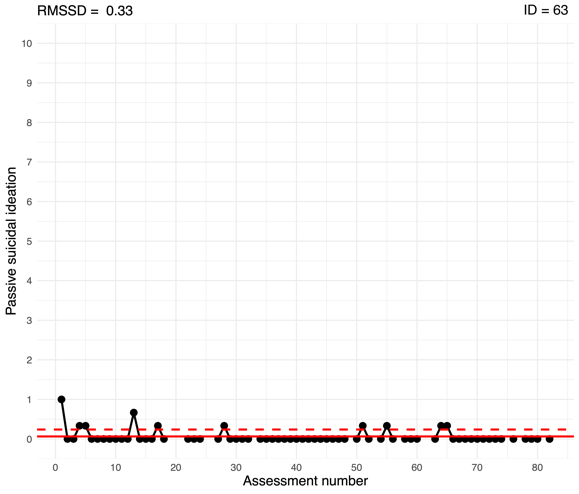

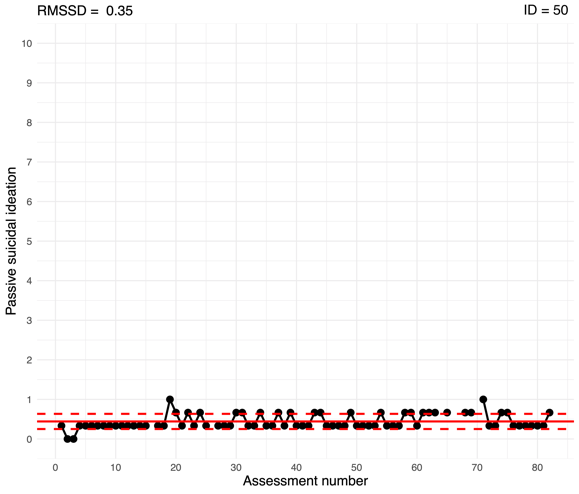

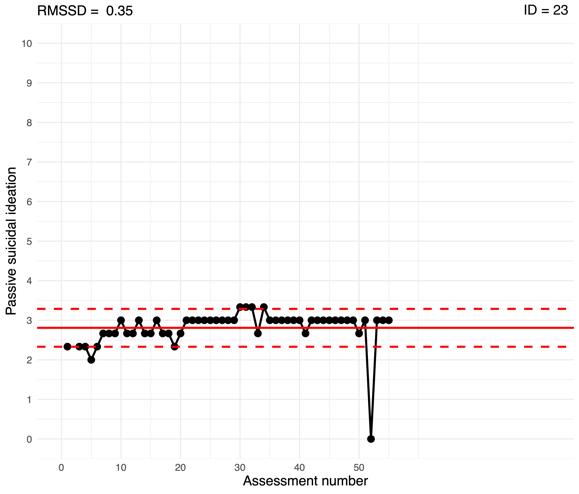

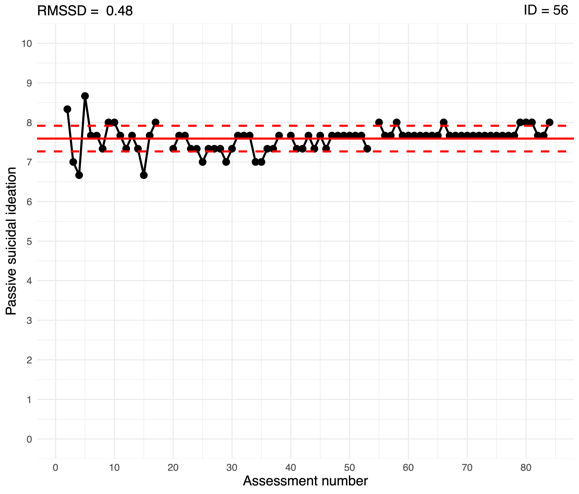

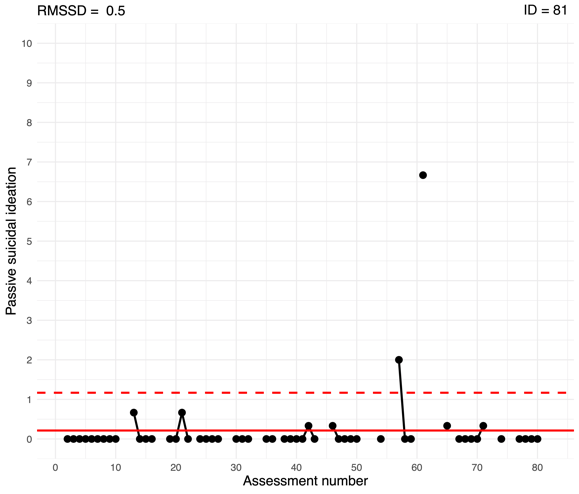

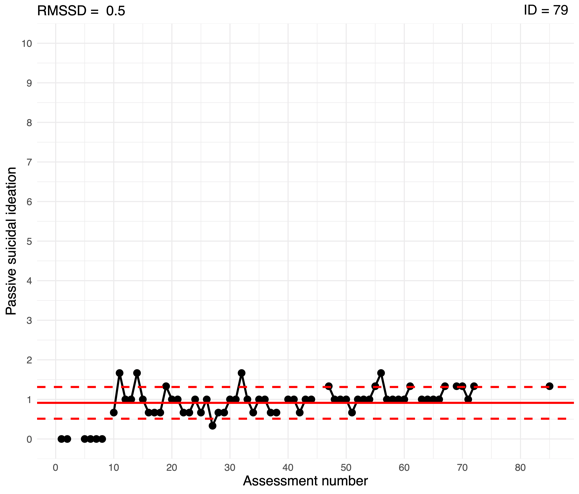

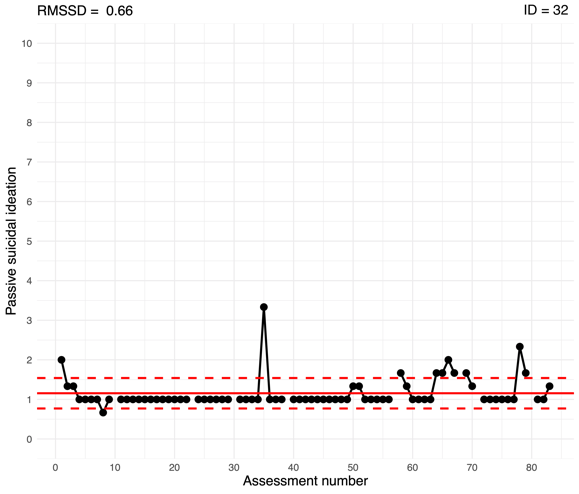

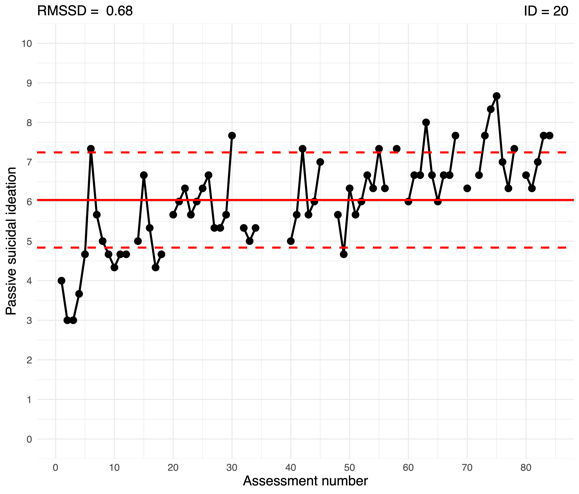

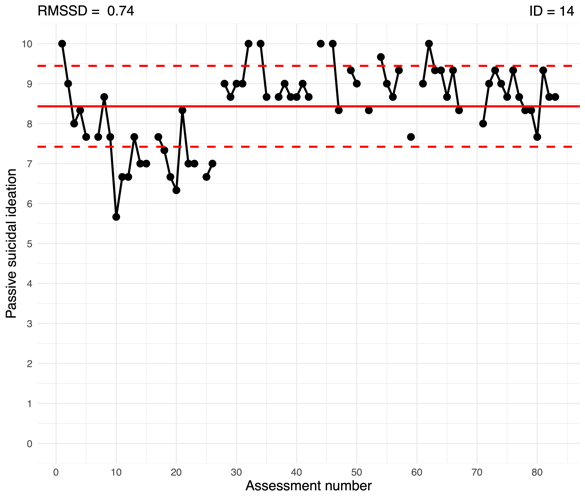

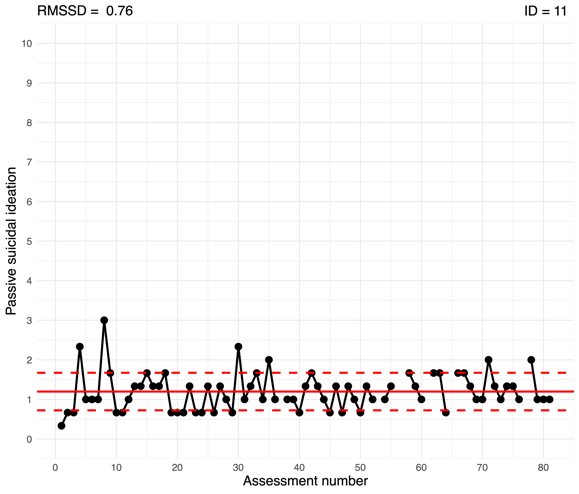

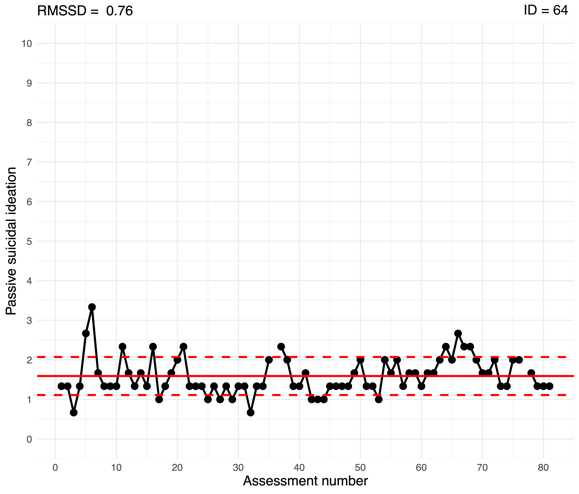
**

**
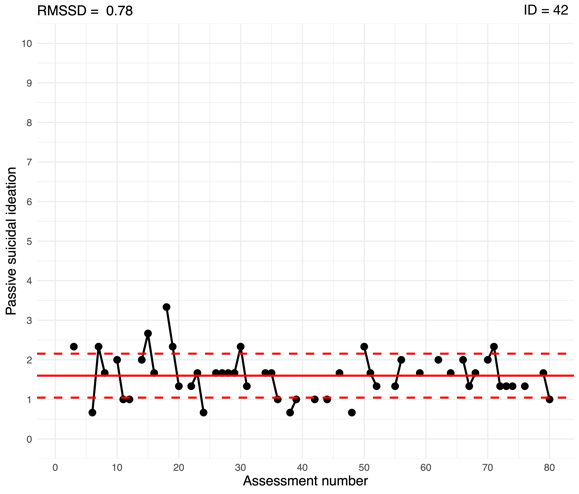

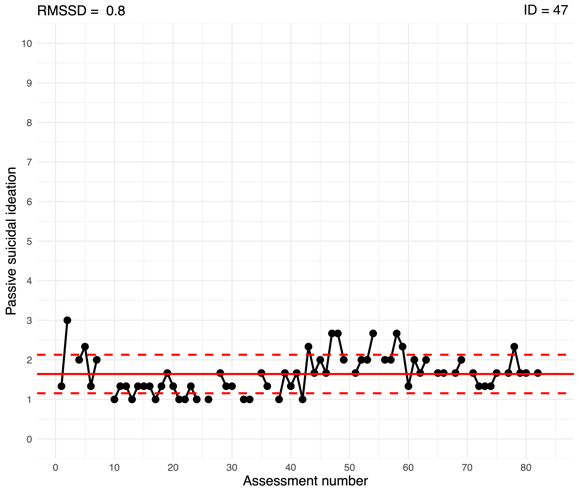

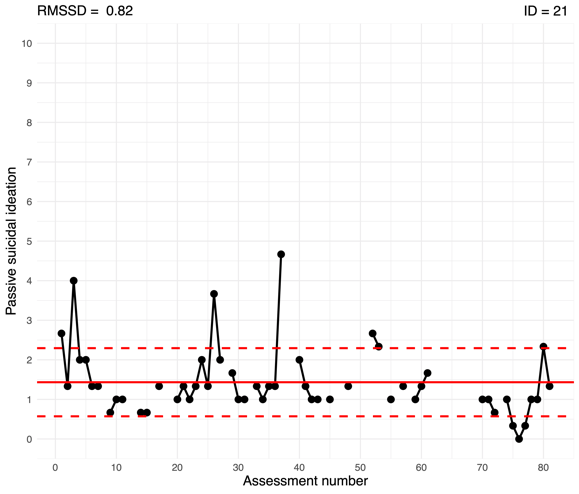

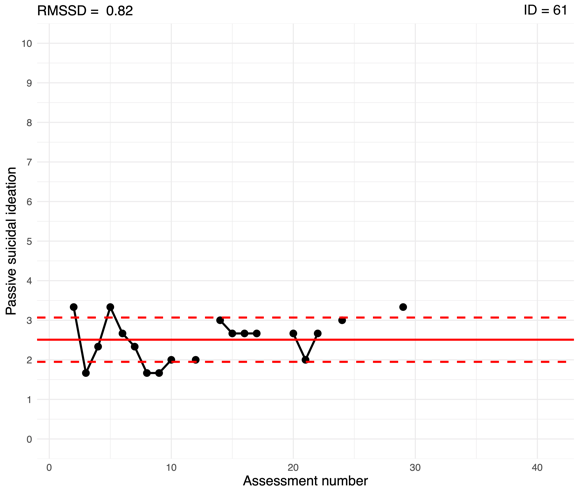

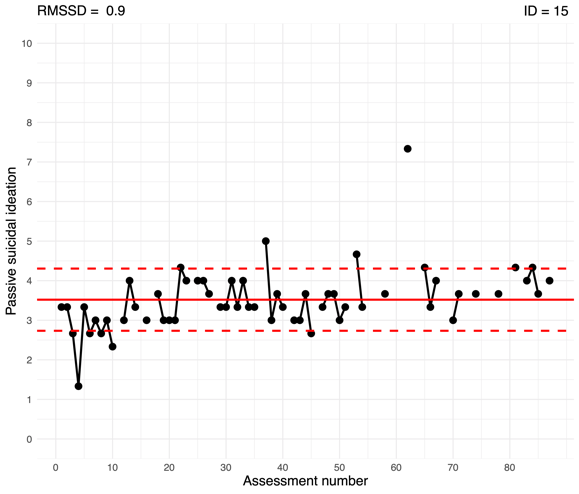

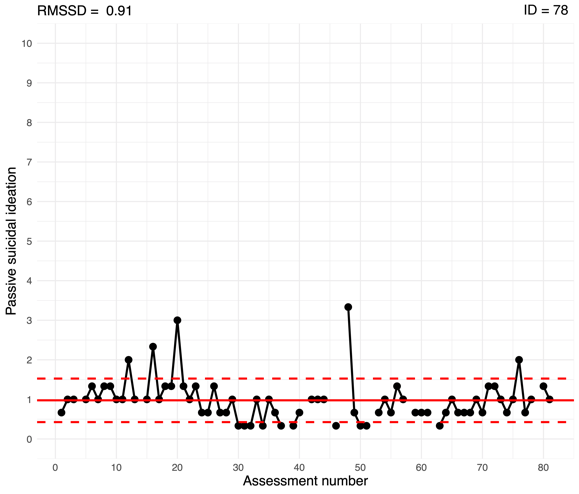

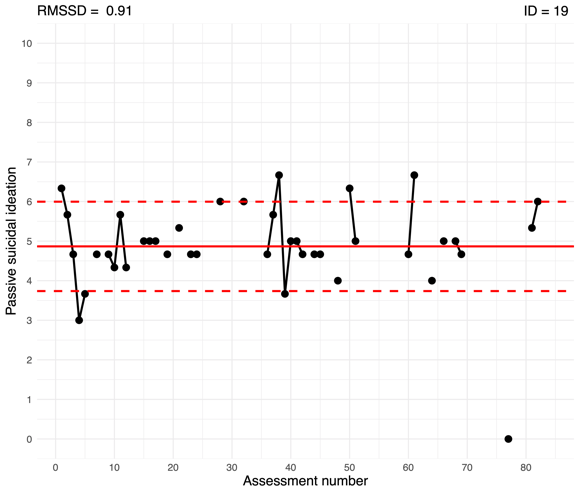

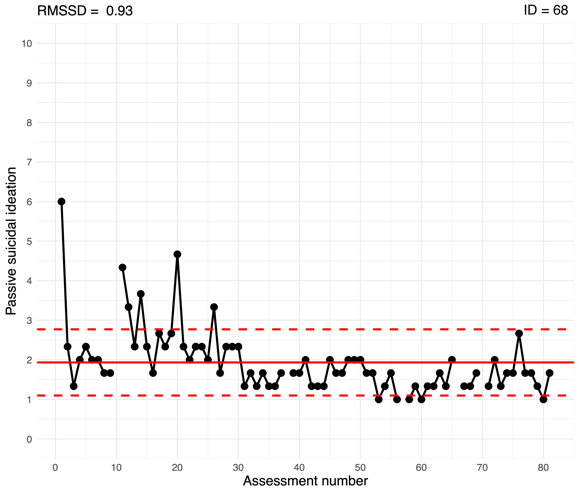

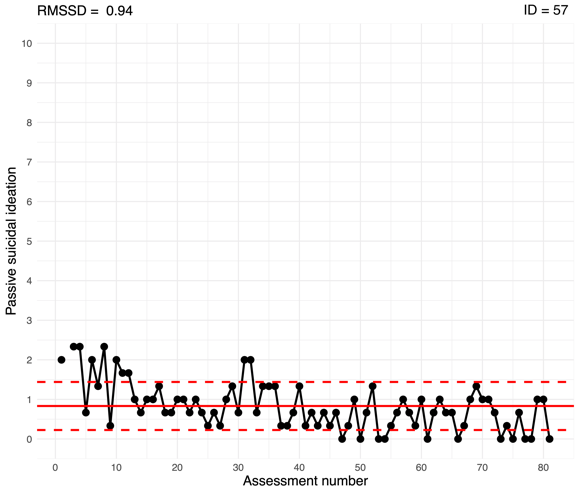

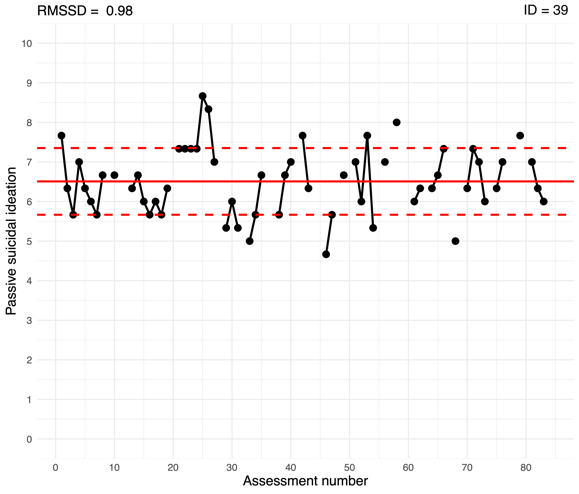

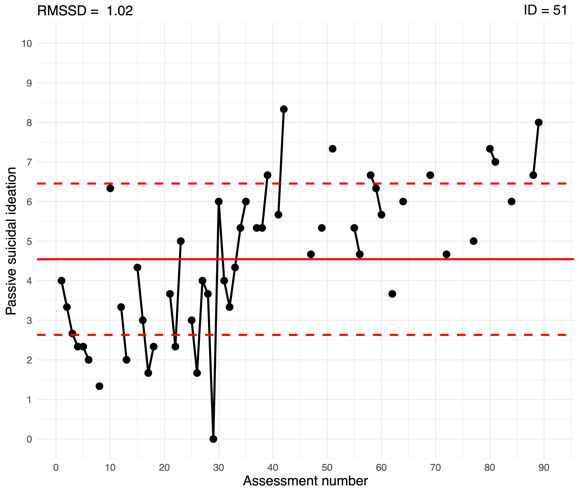

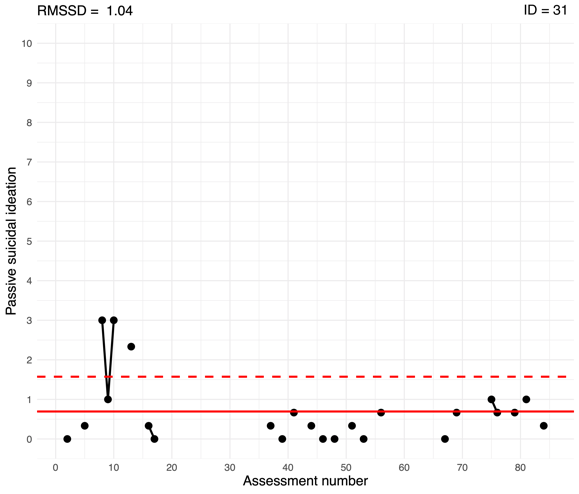

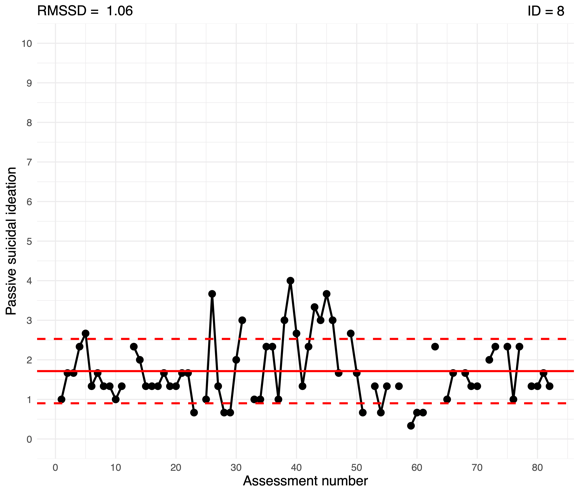

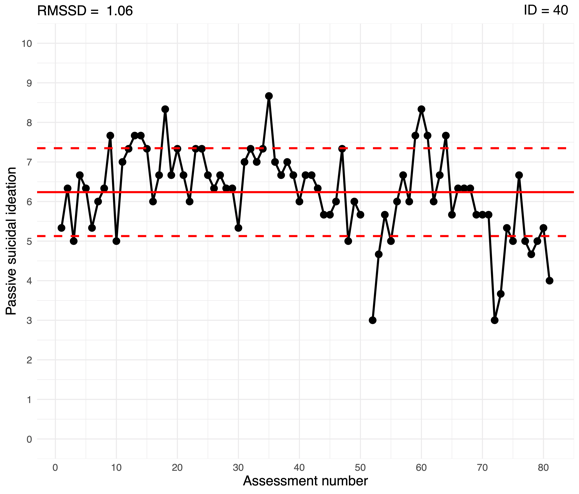

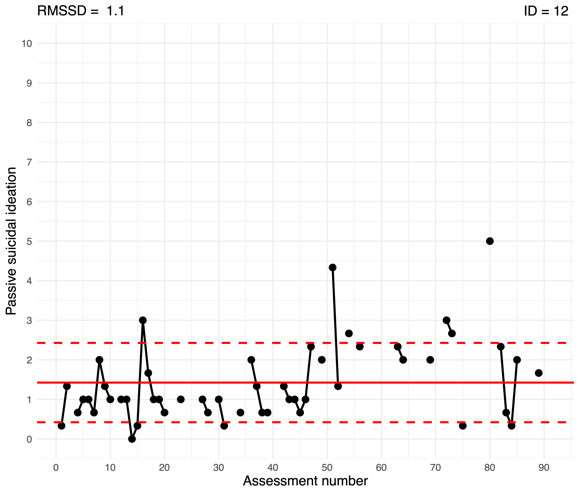

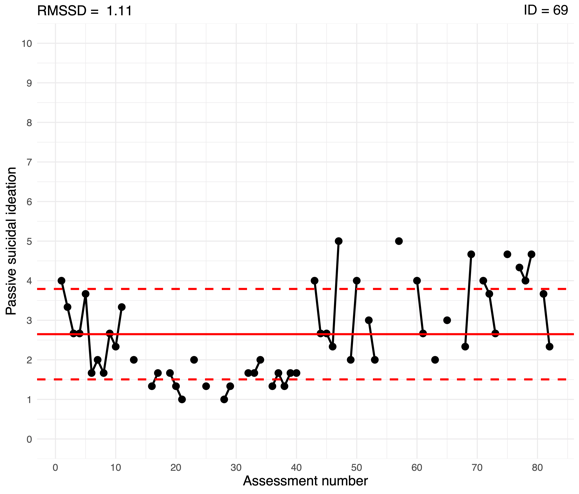

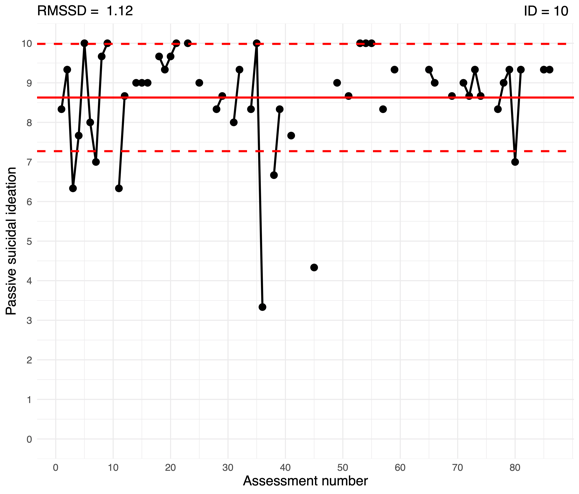

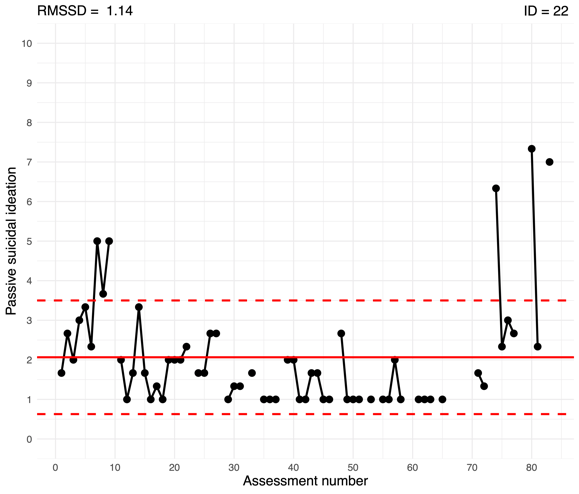

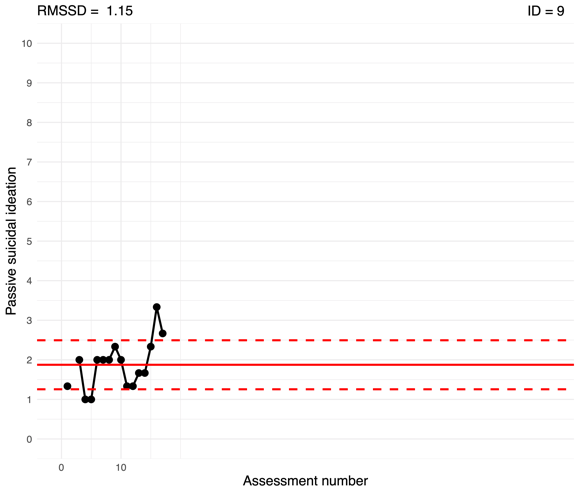

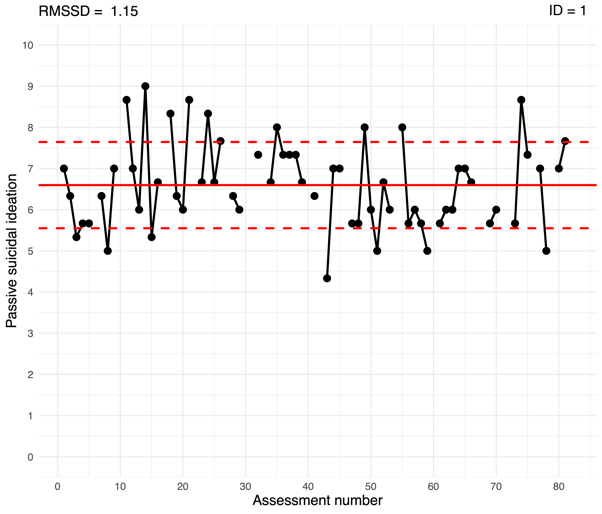

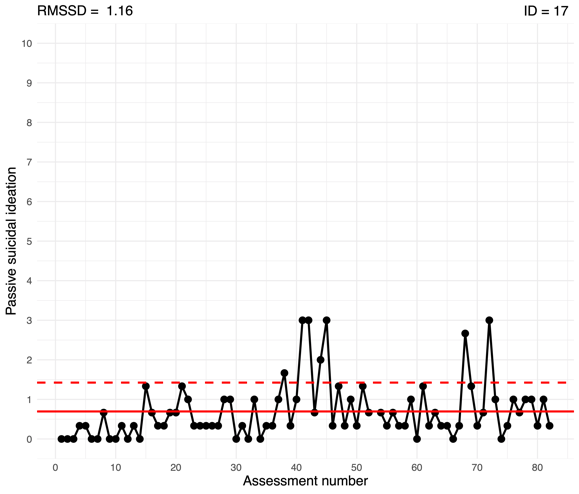

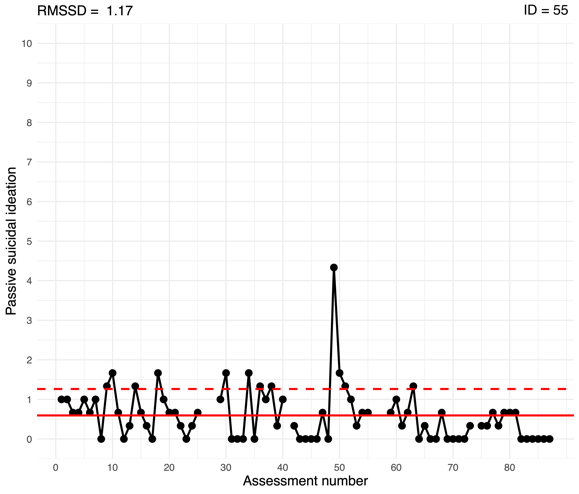

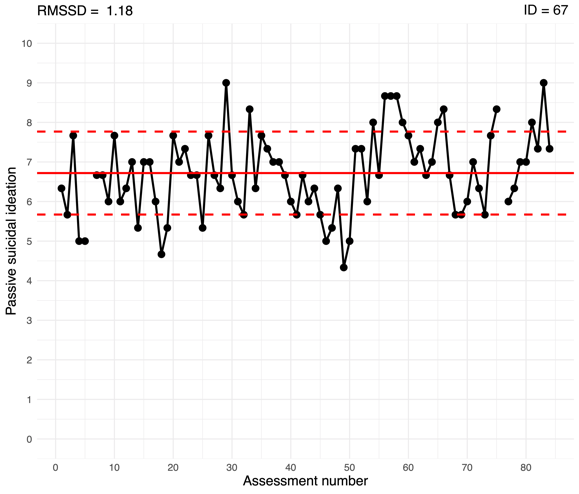

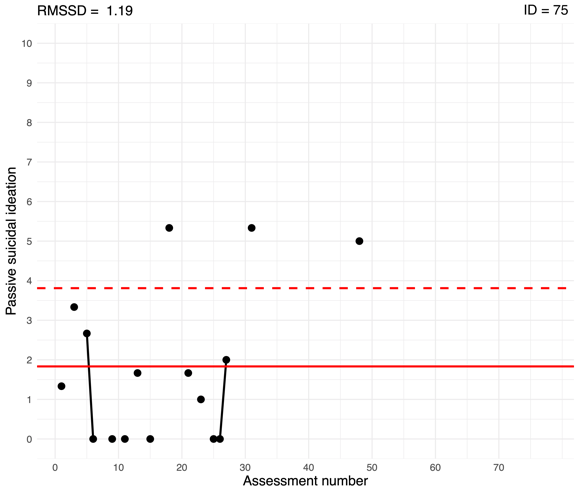

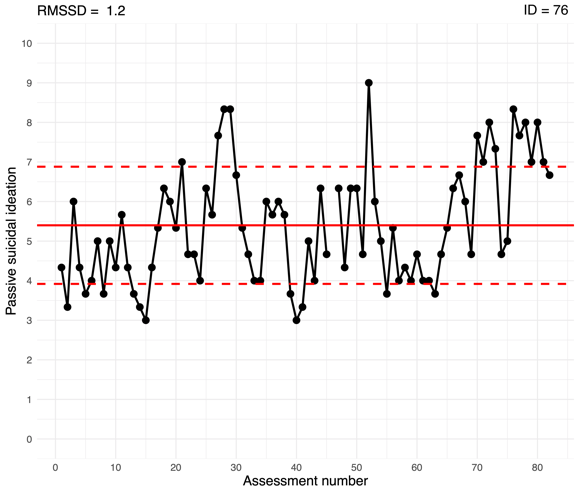

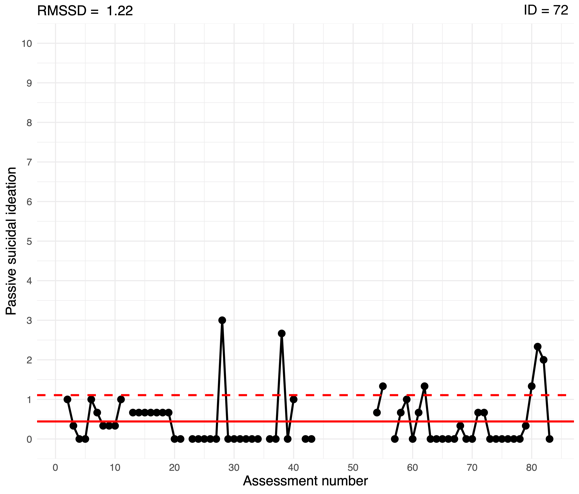

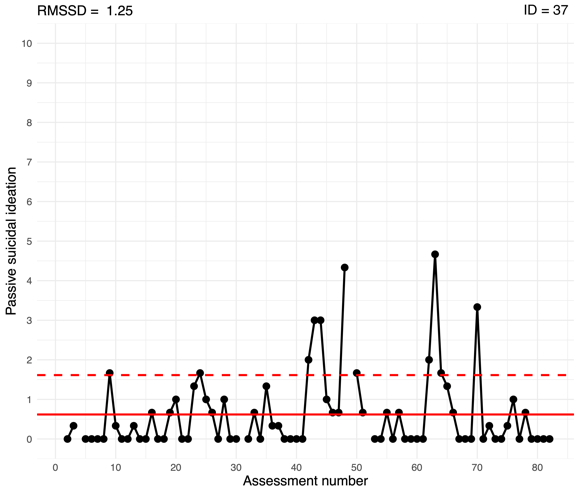

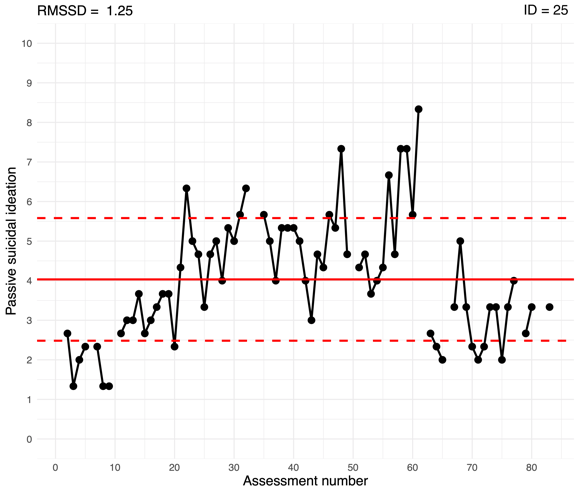

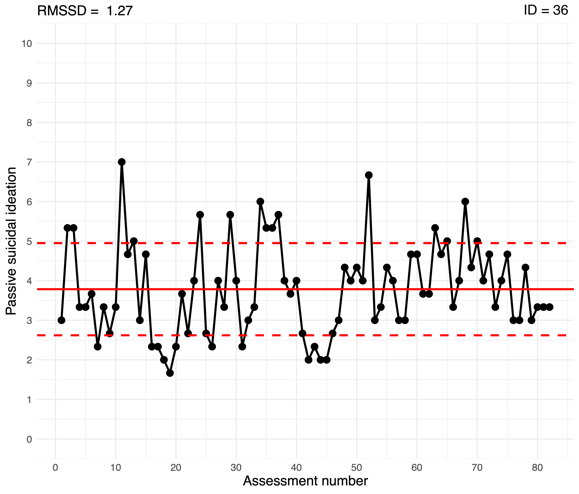

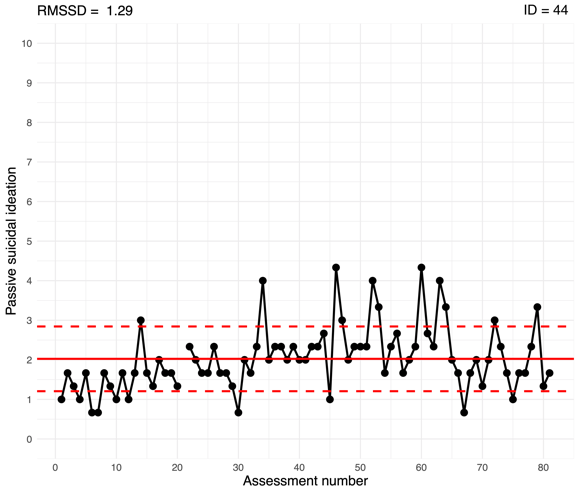

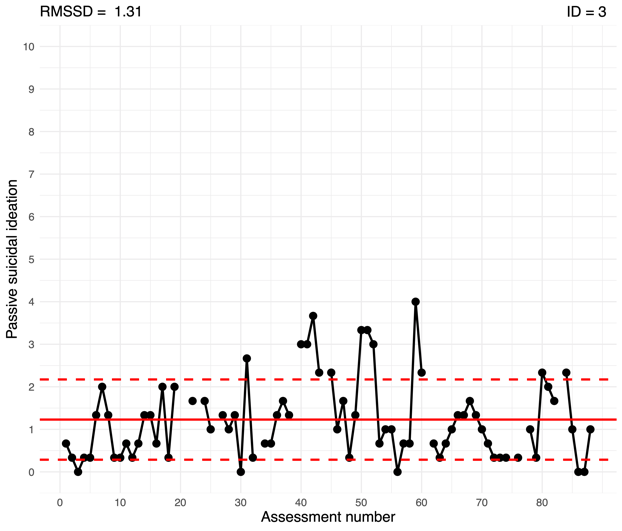

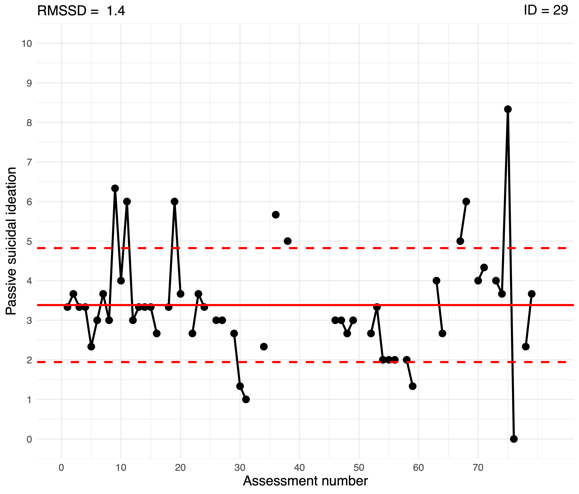

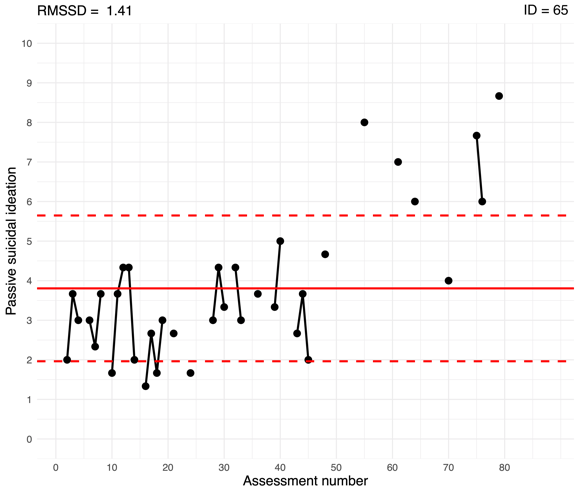

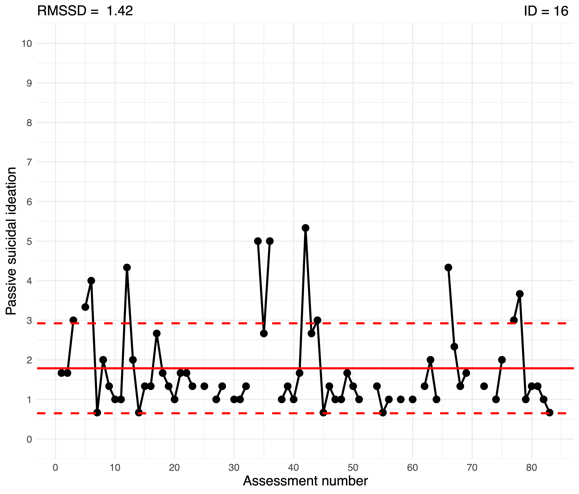

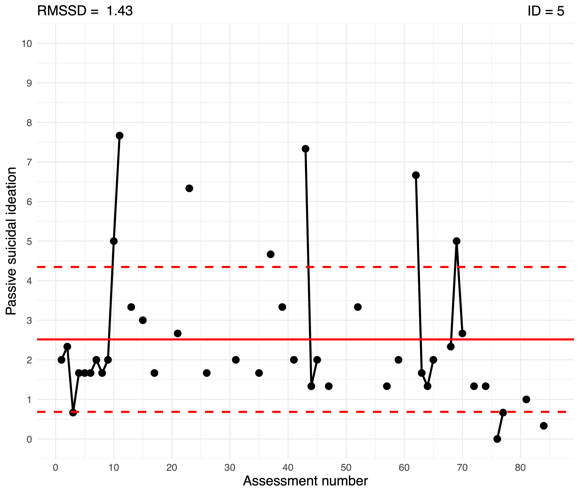

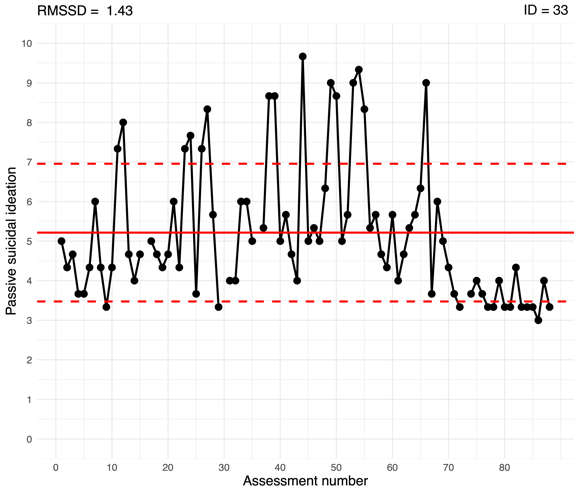

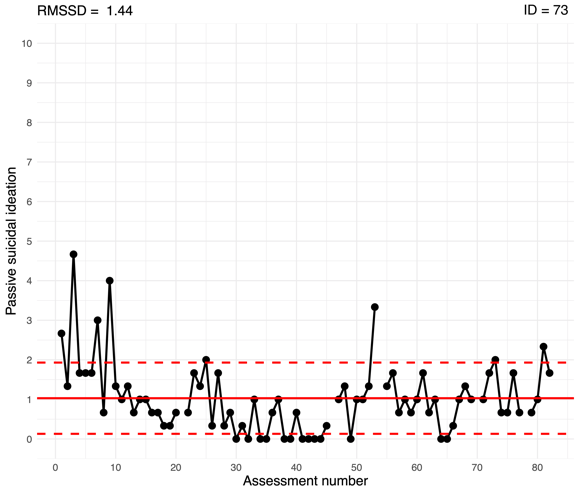

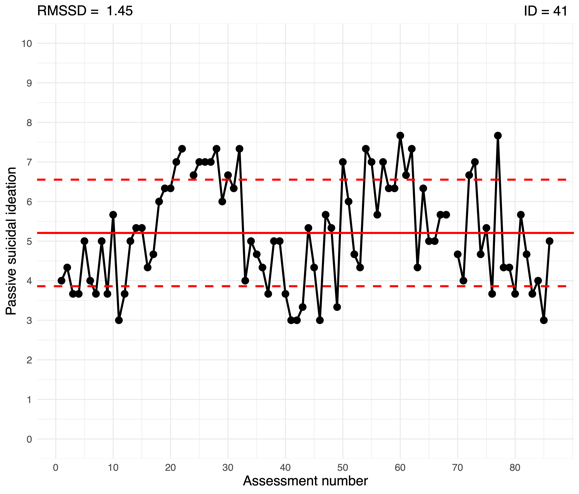

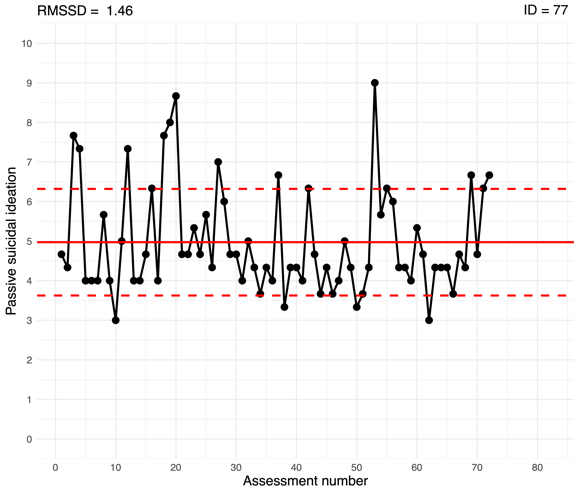

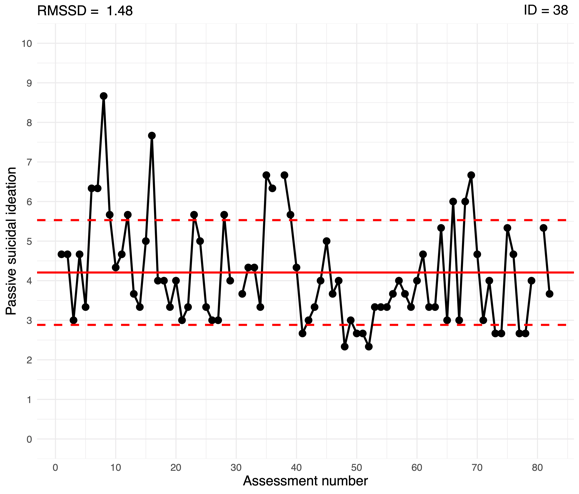

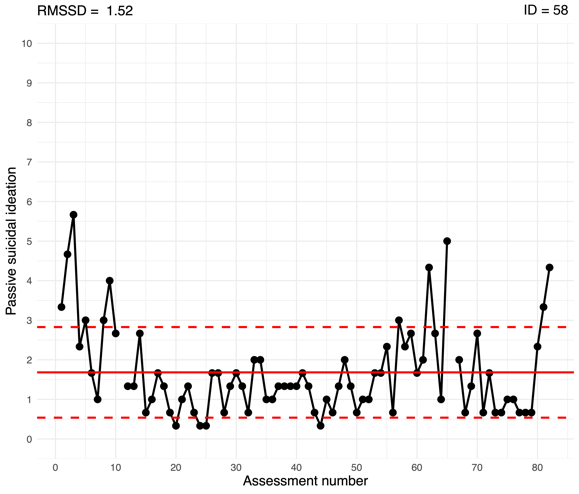

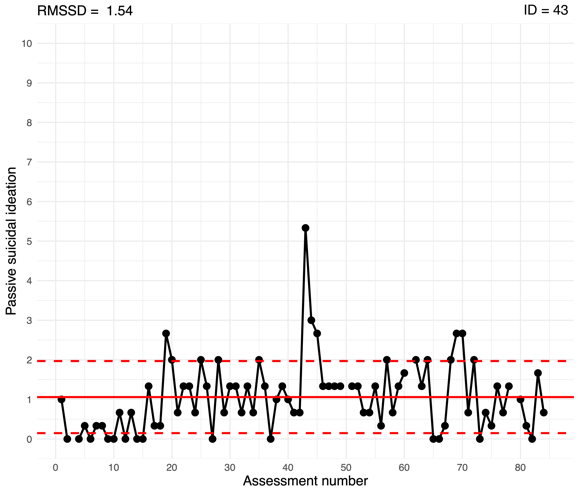

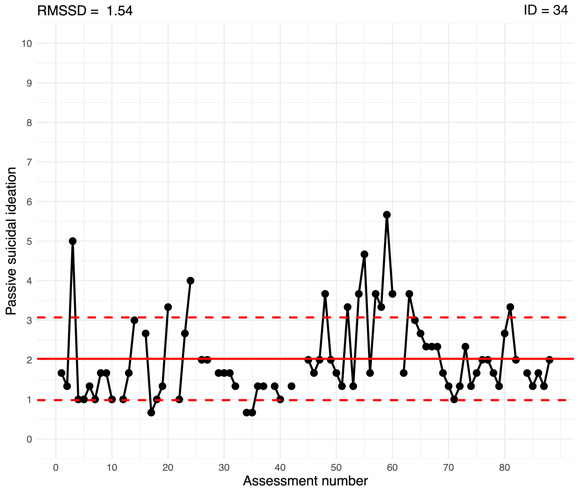

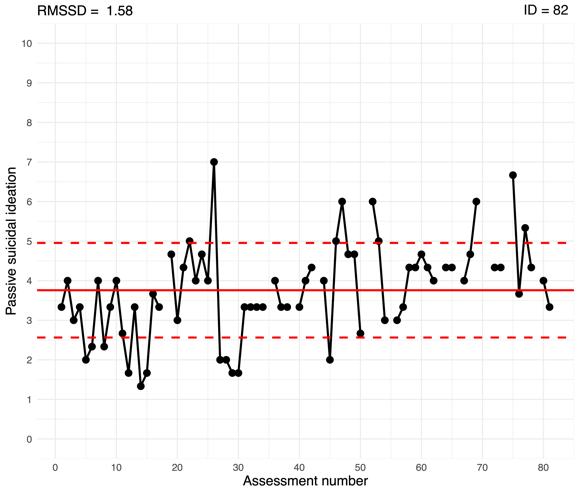

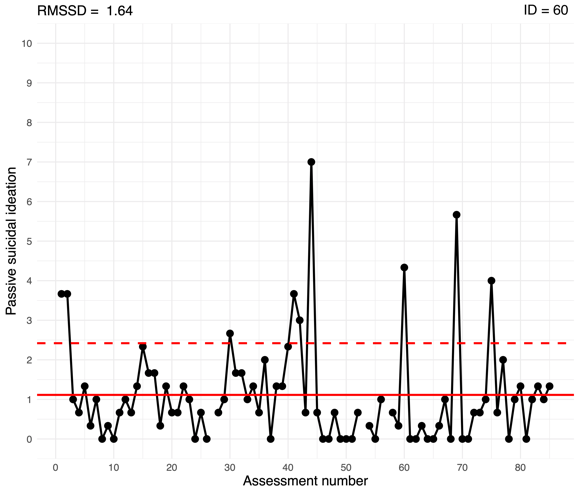

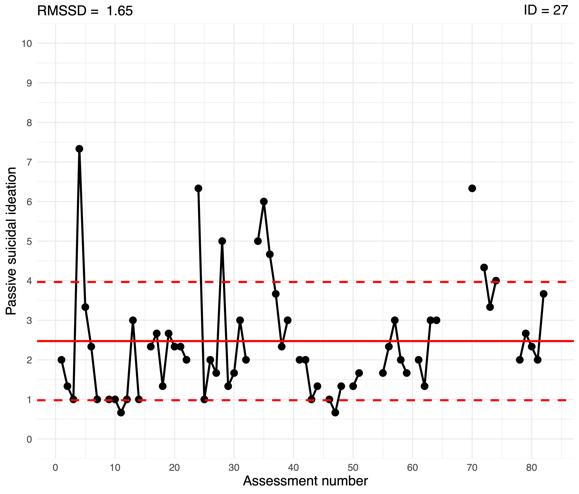

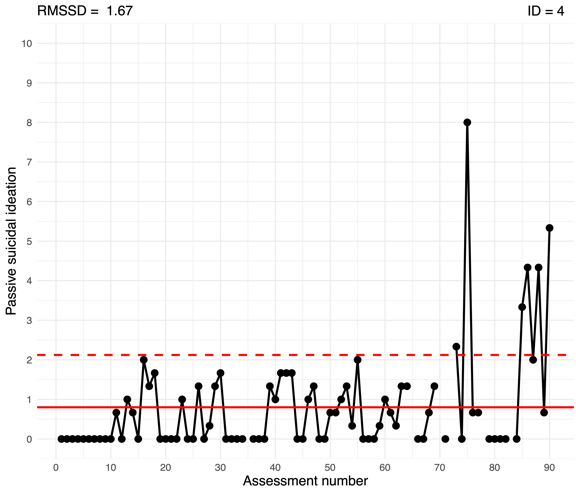

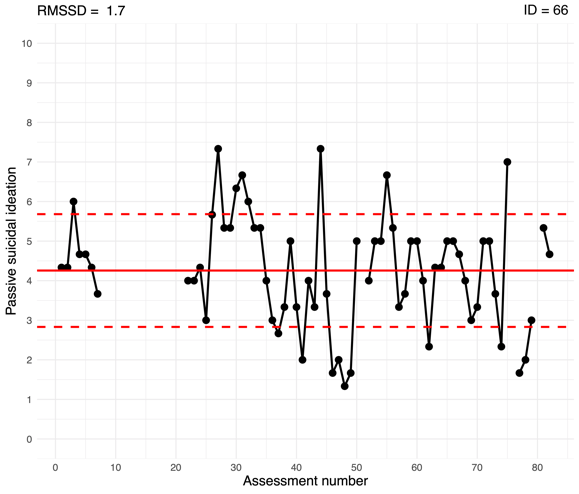

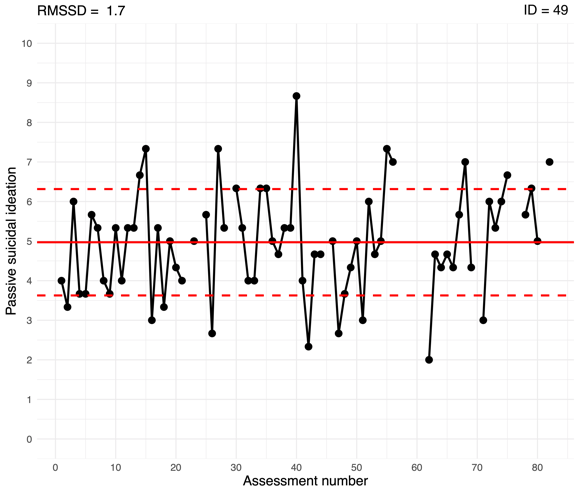

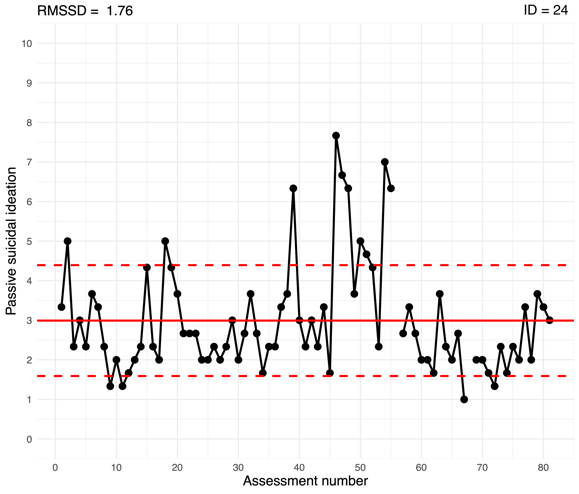

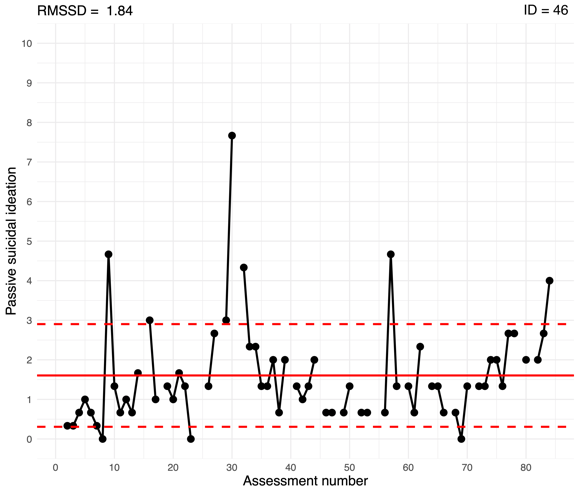

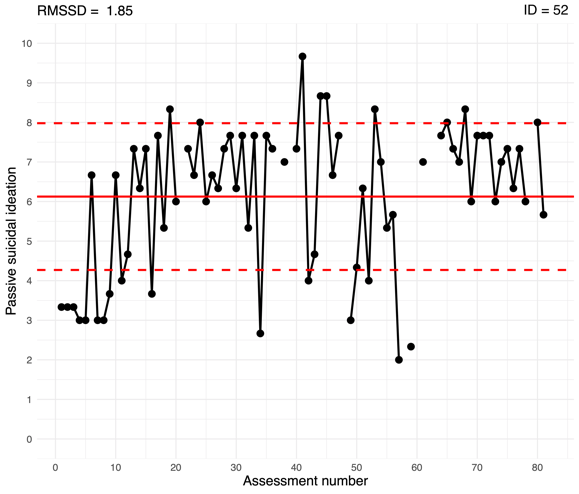

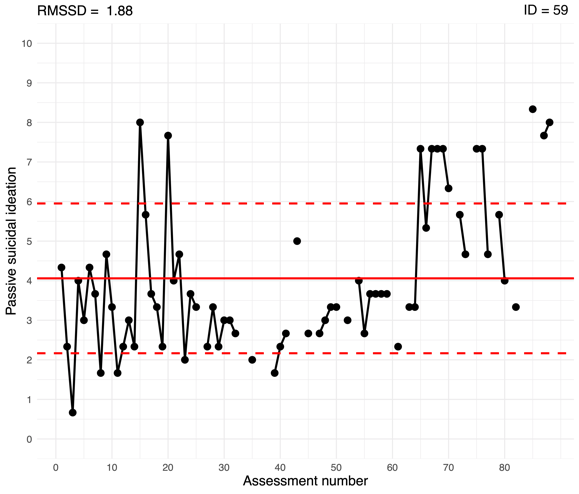

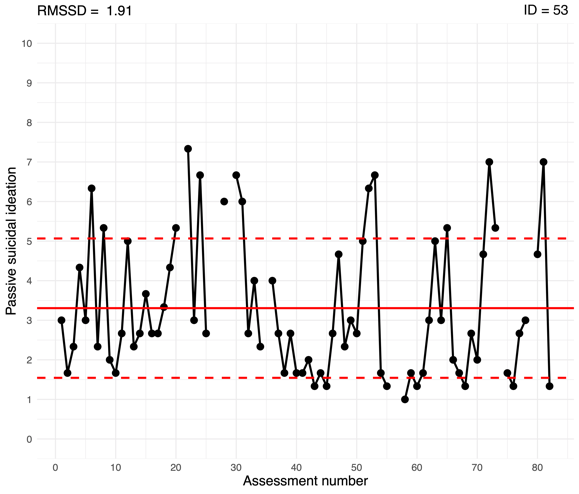

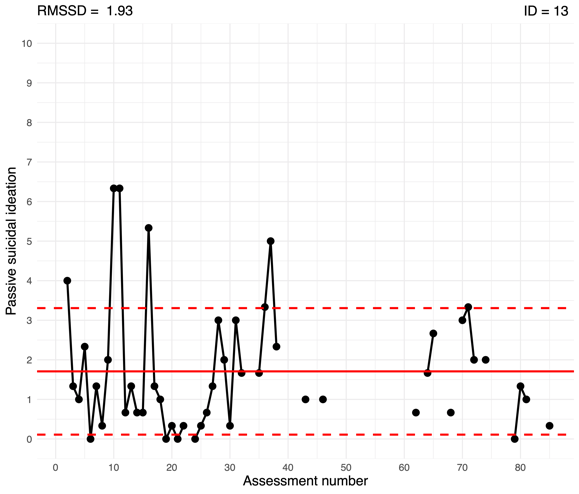

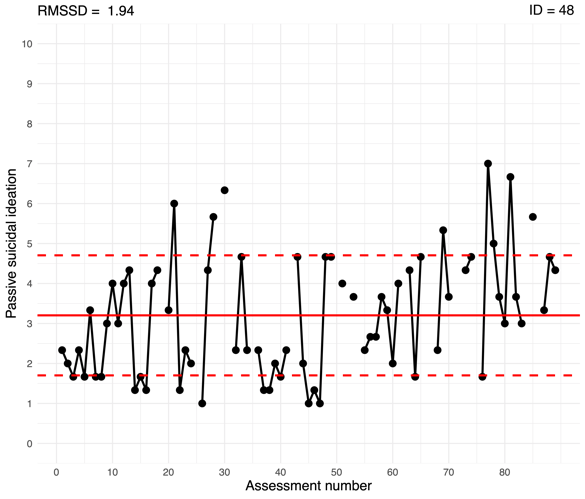

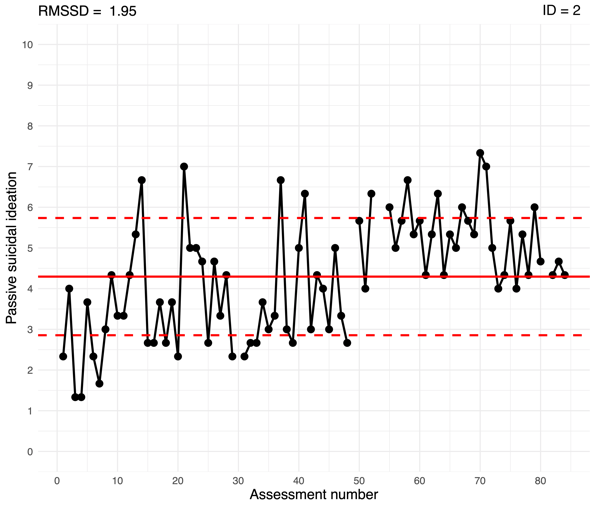

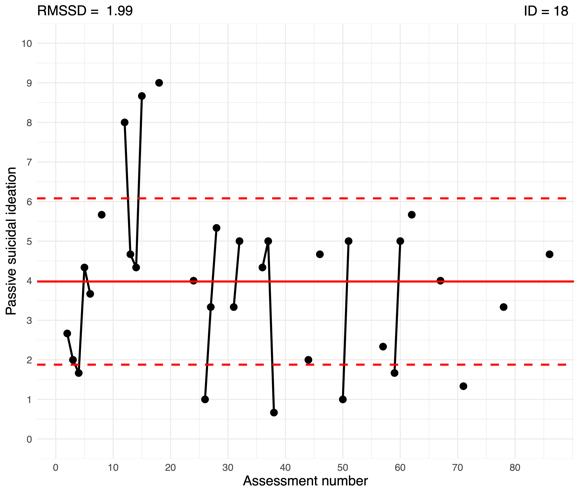

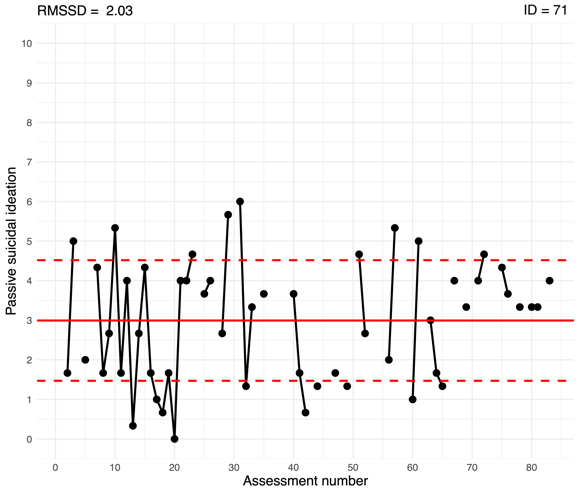

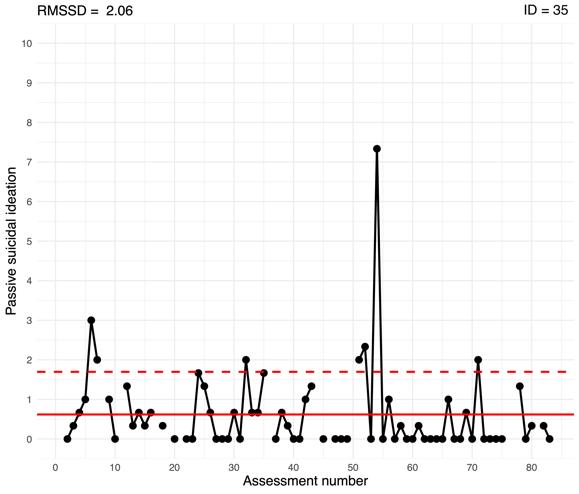

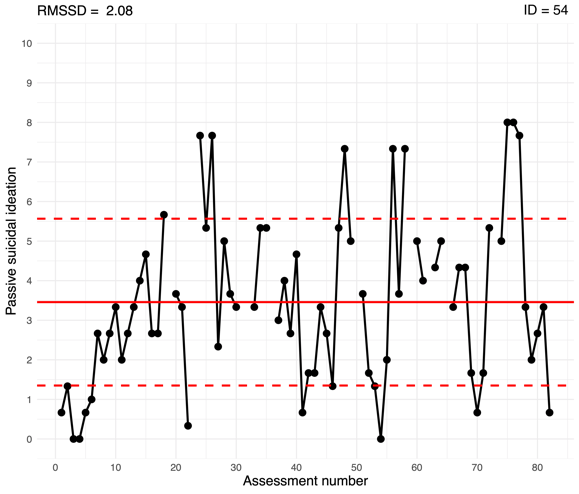

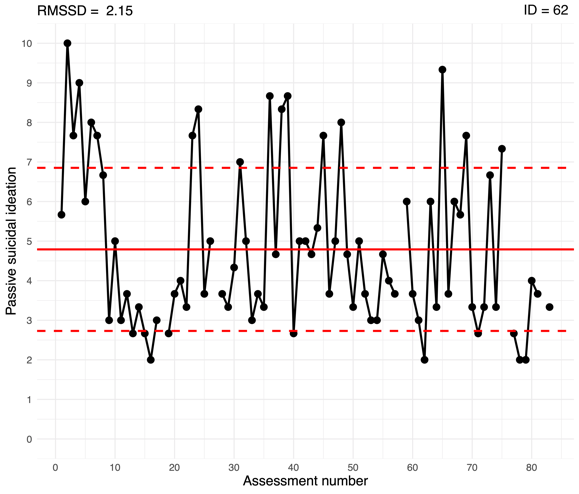

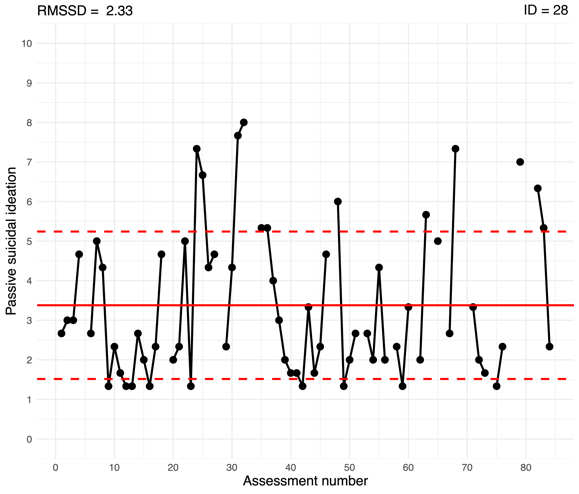

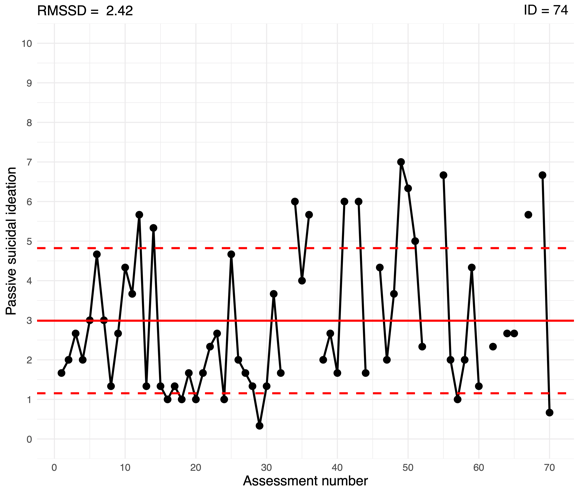

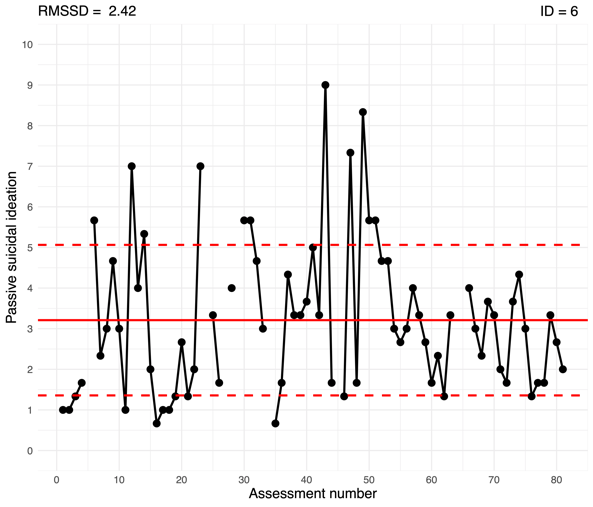

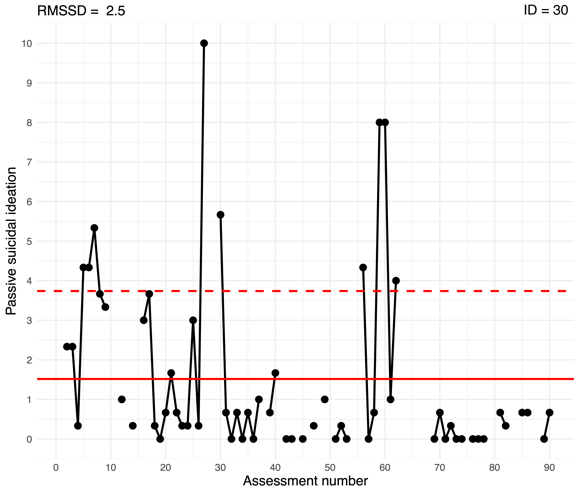

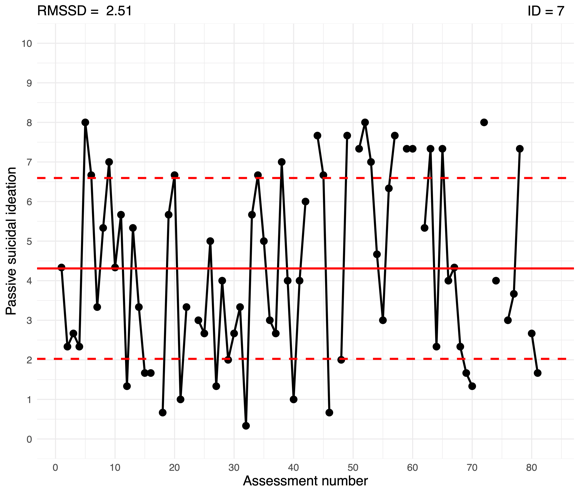

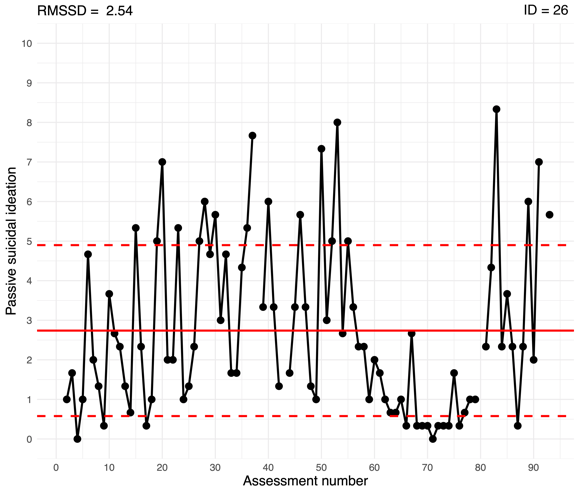

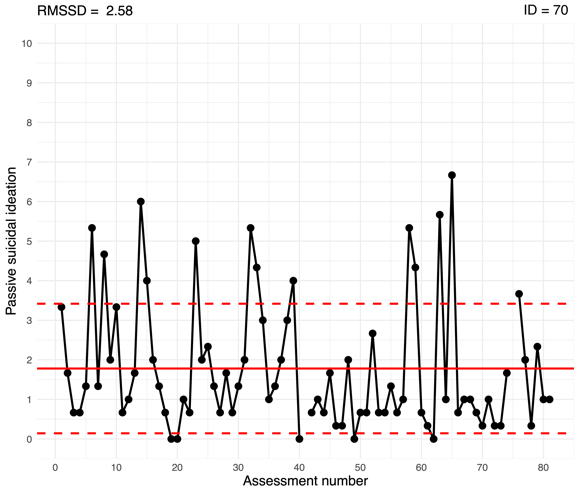

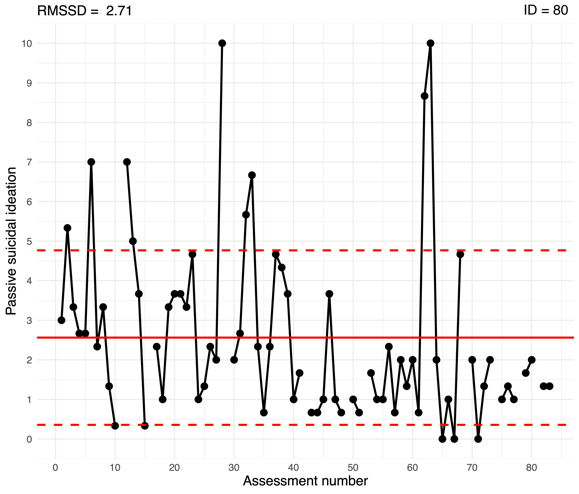
**

*Note:* Time-series plots are presented in order of low to high RMSSD (root mean square of successive differences). The solid red line indicates the person-mean, and dashed red lines the standard deviation around the mean. Phenotype 1 is represented in red, Phenotype 2 in blue, and Phenotype 3 in green. ID numbers do *not* correspond to participant numbers assigned during data collection.

**Figure S2.** Variability in active suicidal ideation


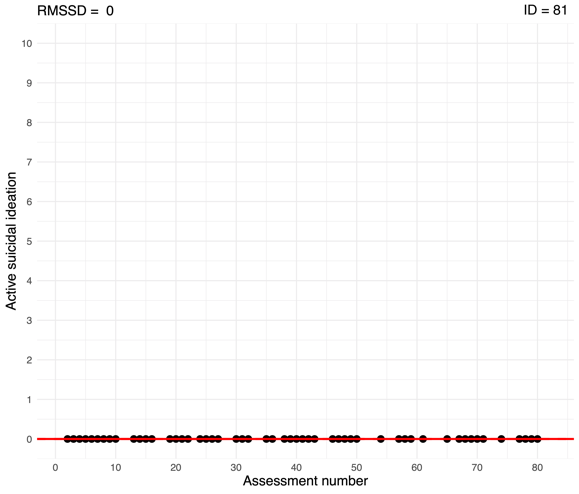

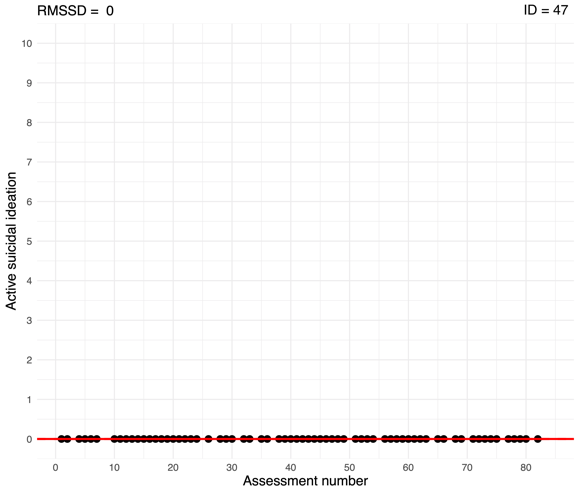

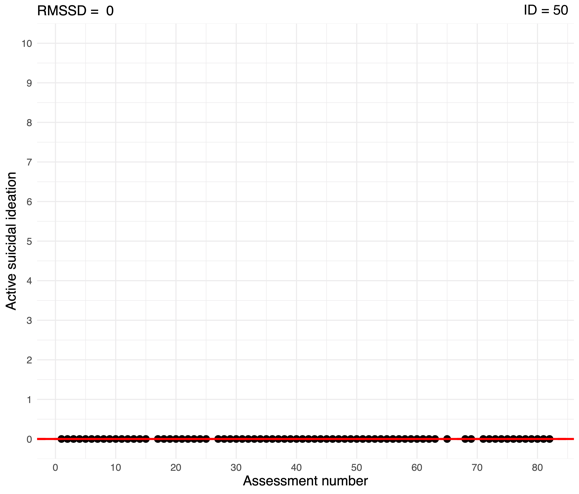

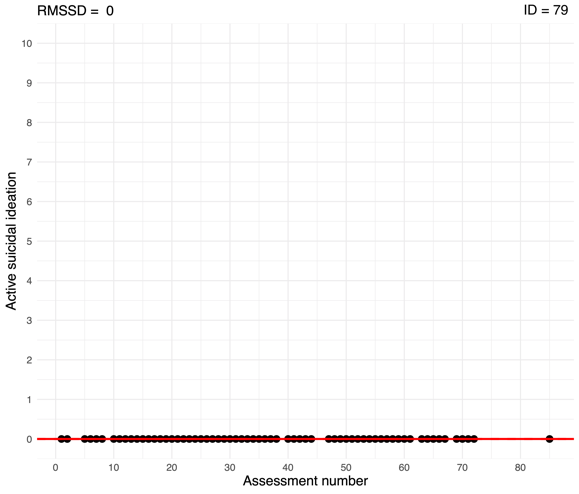

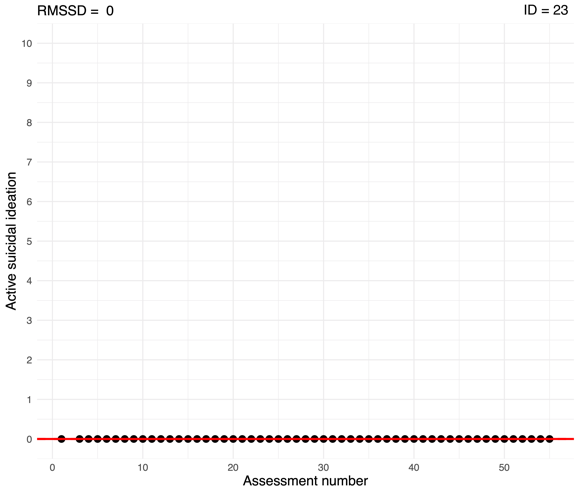

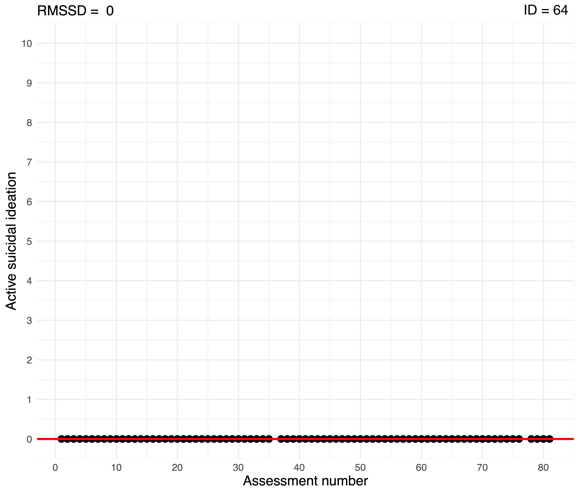

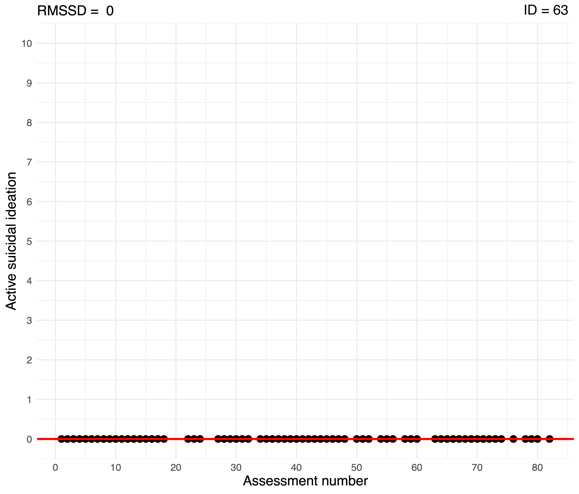

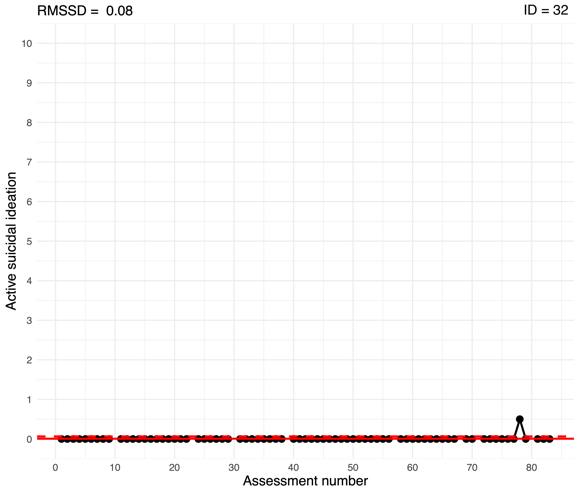

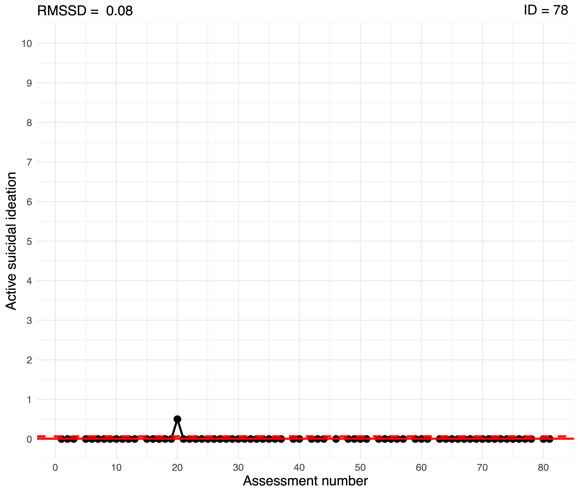

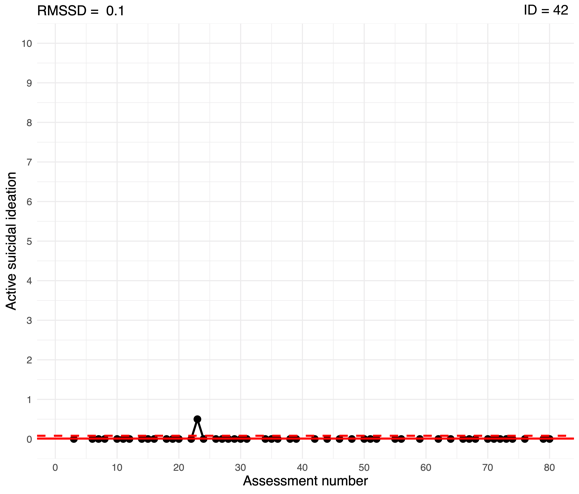

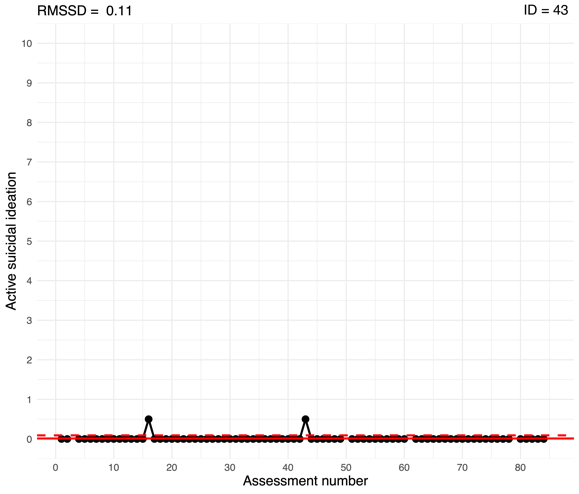

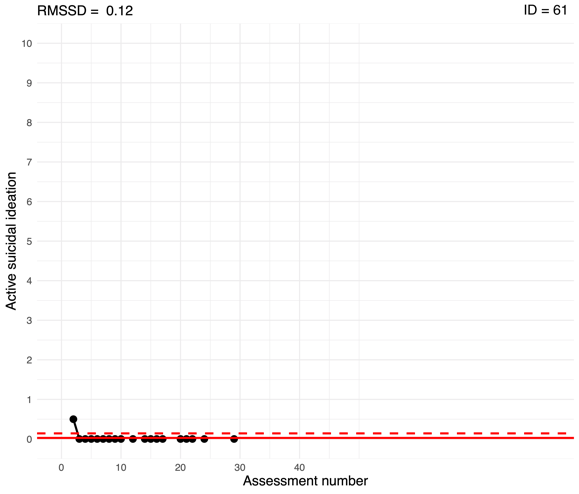

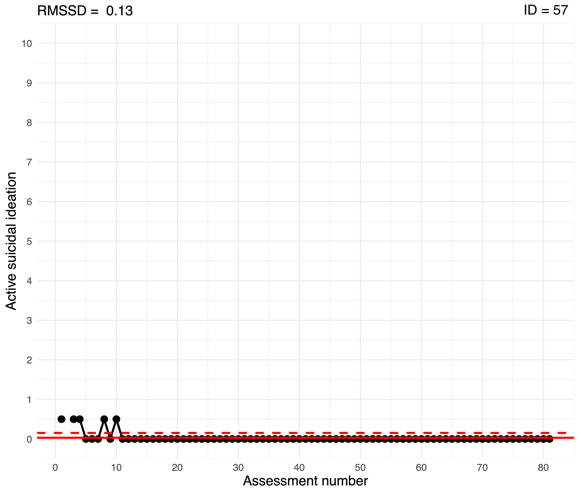

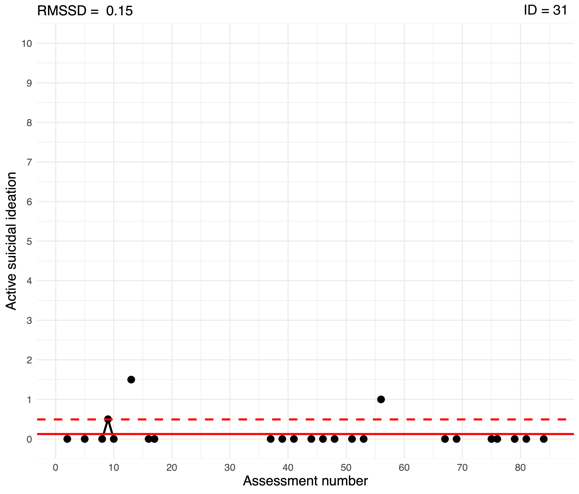

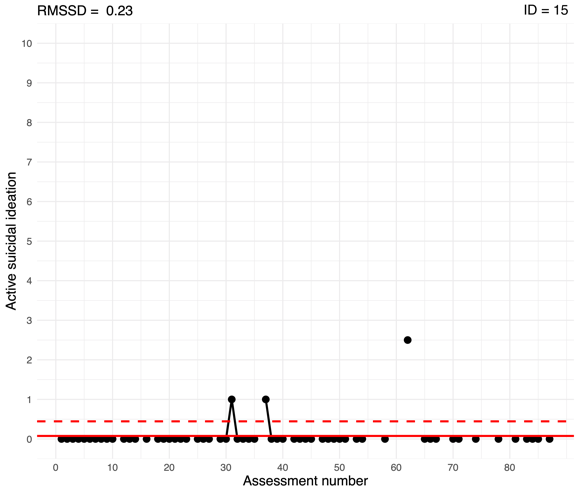

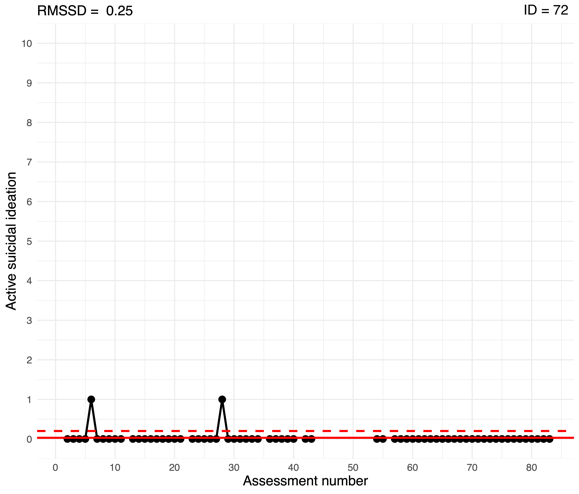

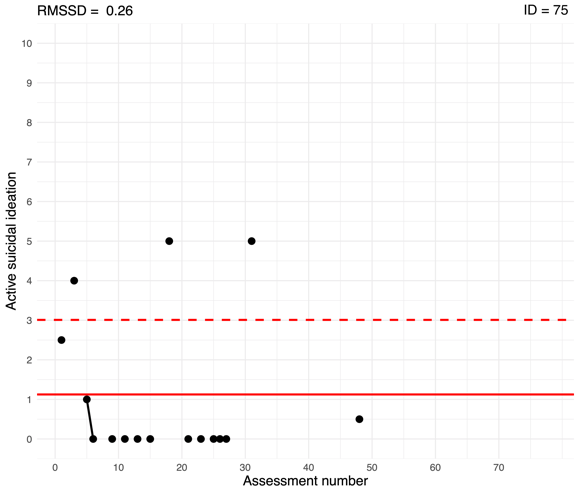

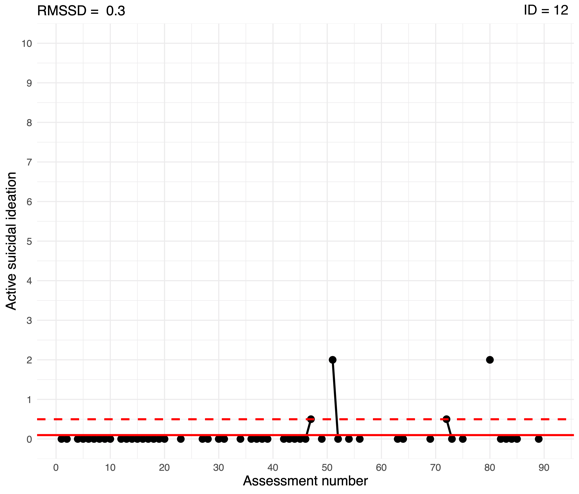


*Note:* Time-series plots are presented in order of low to high RMSSD (root mean square of successive differences). The solid red line indicates the person-mean, and dashed red lines the standard deviation around the mean. Phenotype 1 is represented in red, Phenotype 2 in blue, and Phenotype 3 in green. ID numbers do *not* correspond to participant numbers assigned during data collection.

**Table S1.** Pearson correlations and reliability statistics for the subscales of passive and active

suicidal ideation

|  | 1. | 2. | 3. | 4. | ICC | Cronbach’s alpha |
| --- | --- | --- | --- | --- | --- | --- |
| **Passive suicidal ideation** |  |  |  |  | .70 | 0.85 |
| 1. Desire to live | **-** | **.75** | **.64** | **.54** |  | - |
| 2. Desire to die | **-** | - | **.83** | **.73** |  | - |
| **Active suicidal ideation** |  |  |  |  | .67 | 0.97 |
| 3. Suicidal thoughts | **.64** | **.83** | **-** | **-** |  | - |
| 4. Suicidal intent | **.54** | **.73** | **.94** | **-** |  | - |

*Note:* ICC = Intra-class correlation coefficient. Correlation coefficients significant with *p* < .05

are indicated in **bold**.

**Table S2.** Pearson correlations between passive and active suicidal ideation characteristics

|  | 1. | 2. | 3. | 4. | 5. | 6. | 7. | 8. | 9. |
| --- | --- | --- | --- | --- | --- | --- | --- | --- | --- |
| 1. M, Passive | - | - | - | - | - | - | - | - | - |
| 2. M, Active | **.86** | - | - | - | - | - | - | - | - |
| 3. SD, Passive | **.33** | .13 | - | - | - | - | - | - | - |
| 4. SD, Active | **.62** | **.51** | **.78** | - | - | - | - | - | - |
| 5. Peak, Passive | **.73** | **.56** | **.75** | **.77** | - | - | - | - | - |
| 6. Peak, Active | **.70** | **.68** | **.65** | **.88** | **.84** | - | - | - | - |
| 7. % non-zero, Passive | **.51** | **.27** | **.23** | **.26** | **.28** | **.23** | - | - | - |
| 8. % non-zero, Active | **.82** | **.84** | **.29** | **.60** | **.60** | **.65** | **.36** | - | - |
| 9. RMSSD, Passive | .10 | –.05 | **.82** | **.49** | **.55** | **.43** | **.22** | .06 | - |
| 10. RMSSD, Active | **.55** | **.43** | **.70** | **.90** | **.74** | **.83** | **.26** | **.50** | **.57** |

*Note:* M = Mean, SD = Standard deviation, RMMSD = Root mean square of successive differences; Correlation coefficients significant with *p* < .05 are indicated in **bold**.

**Table S3.** Individual class probabilities for the final three profile solution

|  | **Type 1** | **Type 2** | **Type 3** |  |  | **Type 1** | **Type 2** | **Type 3** |
| --- | --- | --- | --- | --- | --- | --- | --- | --- |
| ID 1 | **1.00e+00** | 2.27e-26 | 3.36e-157 |  | ID 42 | 1.03e-38 | 6.76e-06 | **1.00e+00** |
| ID 2 | 3.39e-20 | **1.00e+00** | 4.08e-06 |  | ID 43 | 1.94e-279 | 4.22e-06 | **1.00e+00** |
| ID 3 | 2.17e-61 | 1.55e-03 | **9.98e-01** |  | ID 44 | 1.87e-35 | 6.67e-04 | **9.99e-01** |
| ID 4 | 0.00e+00 | 6.64e-11 | **1.00e+00** |  | ID 45 | 0.00e+00 | 3.38e-35 | **1.00e+00** |
| ID 5 | 1.10e-17 | **1.00e+00** | 1.22e-11 |  | ID 46 | 1.44e-52 | 3.07e-02 | **9.69e-01** |
| ID 6 | 2.17e-28 | **9.99e-01** | 8.02e-04 |  | ID 47 | 5.46e-40 | 2.72e-06 | **1.00e+00** |
| ID 7 | 1.24e-06 | **1.00e+00** | 4.61e-28 |  | ID 48 | 5.35e-14 | **9.98e-01** | 1.54e-03 |
| ID 8 | 6.01e-29 | 1.61e-04 | **1.00e+00** |  | ID 49 | **1.00e+00** | 1.56e-12 | 1.01e-54 |
| ID 9 | **1.00e+00** | 8.64e-13 | 4.22e-25 |  | ID 50 | 4.64e-50 | 8.09e-13 | **1.00e+00** |
| ID 10 | **1.00e+00** | 4.25e-105 | 0.00e+00 |  | ID 51 | 3.71e-02 | **9.63e-01** | 9.16e-35 |
| ID 11 | 3.50e-33 | 1.55e-08 | **1.00e+00** |  | ID 52 | **1.00e+00** | 1.69e-13 | 5.74e-91 |
| ID 12 | 1.69e-37 | 1.46e-02 | **9.85e-01** |  | ID 53 | 7.19e-19 | **1.00e+00** | 6.82e-07 |
| ID 13 | 9.38e-117 | **9.97e-01** | 2.93e-03 |  | ID 54 | 4.66e-40 | **1.00e+00** | 1.04e-10 |
| ID 14 | **1.00e+00** | 1.36e-137 | 0.00e+00 |  | ID 55 | 0.0e+00 | 8.6e-09 | **1.0e+00** |
| ID 15 | 1.70e-34 | 5.60e-04 | **9.99e-01** |  | ID 56 | **1.00e+00** | 1.02e-277 | 0.00e+00 |
| ID 16 | 4.73e-28 | 1.02e-03 | **9.99e-01** |  | ID 57 | 1.77e-186 | 8.81e-08 | **1.00e+00** |
| ID 17 | 2.09e-305 | 7.33e-09 | **1.00e+00** |  | ID 58 | 3.66e-34 | 1.88e-02 | **9.81e-01** |
| ID 18 | 3.91e-15 | **1.00e+00** | 4.62e-13 |  | ID 59 | **1.00e+00** | 7.54e-08 | 5.09e-39 |
| ID 19 | **1.00e+00** | 1.13e-28 | 1.30e-202 |  | ID 60 | 0.00e+00 | 4.34e-12 | **1.00e+00** |
| ID 20 | **1.00e+00** | 9.11e-12 | 1.10e-28 |  | ID 61 | 8.79e-35 | 1.24e-04 | **1.00e+00** |
| ID 21 | 2.38e-35 | 1.26e-05 | **1.00e+00** |  | ID 62 | 2.77e-11 | **1.00e+00** | 7.91e-08 |
| ID 22 | 3.98e-32 | **1.00e+00** | 2.42e-04 |  | ID 63 | 0.00e+00 | 1.16e-32 | **1.00e+00** |
| ID 23 | 1.00e-41 | 4.95e-04 | **1.00e+00** |  | ID 64 | 2.97e-40 | 3.03e-06 | **1.00e+00** |
| ID 24 | **1.00e+00** | 2.44e-10 | 7.65e-27 |  | ID 65 | 2.66e-25 | **1.00e+00** | 6.47e-25 |
| ID 25 | 6.59e-23 | **1.00e+00** | 2.68e-06 |  | ID 66 | **1.00e+00** | 2.26e-09 | 4.80e-26 |
| ID 26 | 2.52e-28 | **1.00e+00** | 9.68e-08 |  | ID 67 | **1.00e+00** | 2.07e-85 | 0.00e+00 |
| ID 27 | **1.00e+00** | 1.11e-05 | 9.42e-15 |  | ID 68 | 8.34e-10 | 6.10e-03 | **9.94e-01** |
| ID 28 | 2.30e-26 | **1.00e+00** | 8.51e-08 |  | ID 69 | **1.00e+00** | 1.01e-11 | 6.91e-21 |
| ID 29 | 4.81e-34 | **88e-01** | 16e-02 |  | ID 70 | 3.35e-59 | **9.29e-01** | 7.10e-02 |
| ID 30 | 0.00000 | **0.99859** | 0.00141 |  | ID 71 | 2.23e-33 | **9.99e-01** | 1.18e-03 |
| ID 31 | 0.00e+00 | 2.11e-06 | **1.00e+00** |  | ID 72 | 0.00e+00 | 1.36e-14 | **1.00e+00** |
| ID 32 | 2.44e-40 | 1.56e-07 | **1.00e+00** |  | ID 73 | 2.28e-262 | 9.48e-05 | **1.00e+00** |
| ID 33 | 1.05e-02 | **9.89e-01** | 9.89e-11 |  | ID 74 | 1.33e-17 | **1.00e+00** | 2.07e-07 |
| ID 34 | 3.98e-32 | 1.40e-02 | **9.86e-01** |  | ID 75 | 0.0e+00 | **1.0e+00** | 9.6e-60 |
| ID 35 | 0.00e+00 | 1.86e-14 | **1.00e+00** |  | ID 76 | 1.90e-06 | **1.00e+00** | 2.33e-08 |
| ID 36 | **1.00e+00** | 4.74e-07 | 1.19e-27 |  | ID 77 | **1.00e+00** | 5.83e-29 | 1.80e-193 |
| ID 37 | 0.0e+00 | 1.3e-12 | **1.0e+00** |  | ID 78 | 1.06e-39 | 6.90e-07 | **1.00e+00** |
| ID 38 | 1.62e-10 | **1.00e+00** | 1.07e-05 |  | ID 79 | 2.75e-111 | 7.16e-09 | **1.00e+00** |
| ID 39 | **1.00e+00** | 6.25e-87 | 0.00e+00 |  | ID 80 | 4.48e-34 | **1.00e+00** | 2.45e-05 |
| ID 40 | **1.00e+00** | 4.74e-09 | 3.10e-37 |  | ID 81 | 0.00e+00 | 2.47e-32 | **1.00e+00** |
| ID 41 | **1.00e+00** | 6.89e-33 | 4.16e-215 |  | ID 82 | 6.16e-26 | **9.75e-01** | 2.49e-02 |

*Note:* The class that the participant was ultimately assigned to is indicated in **bold**.

**Figure S3.** Graphical depiction of similarities with the phenotypes by Kleiman et al. (2018)
